# Genome-resolved metagenomics reveals co-selection of antibiotic and metal resistance in chronically polluted industrial soils

**DOI:** 10.3389/fmicb.2026.1829529

**Published:** 2026-05-08

**Authors:** Zhanar Abilda, Iskander Isgandarov, Rakhim Kanat, Dias Daurov, Zagipa Sapakhova, Kabyl Zhambakin, Ainash Daurova, Dinara Begaliyeva, Khanylbek Choi, Malika Shamekova

**Affiliations:** 1 Laboratory of Breeding and Biotechnology, Institute of Plant Biology and Biotechnology, Almaty, Kazakhstan; 2 Tanir Research Laboratory, Almaty, Kazakhstan

**Keywords:** antibiotic resistance genes, heavy metal pollution, long-read sequencing, metagenomics, metal resistance genes, Oxford Nanopore, soil community

## Abstract

**Introduction:**

Chronic heavy metal contamination can restructure soil microbiomes and may co-select for antibiotic resistance, yet genome-resolved evidence from industrial soils remains limited.

**Methods:**

In this study, we applied Oxford Nanopore long-read metagenomic sequencing to soil samples collected across industrially influenced sites in East Kazakhstan to characterize strain-level community composition, profile antibiotic resistance genes and metal resistance genes, and relate these patterns to soil physicochemical properties.

**Results:**

Across all samples, we identified 3,053 strains, with Actinobacteria and Proteobacteria together accounting for 94.1% of the total community. Heavy metal concentrations varied markedly among sites. The resistome comprised antibiotic resistance genes from several drug classes and 238 distinct metal resistant genes, with aminoglycoside, glycopeptide, and multidrug resistance dominating the antibiotic resistance gene profile, while *czcA, ruvB, arsM*, and *arsT* were among the most abundant Metal resistant genes. Multivariate analyses showed that heavy metals, particularly Zn, significantly shaped microbial community structure as well as antibiotic resistance gene and metal resistance gene composition, and redundancy analysis identified Zn and soil pH as the principal environmental drivers. Network analyses further revealed that *Bradyrhizobium icense* and *Conexibacter woesei* acted as key super-hosts linking ARGs and MRGs, supporting heavy metal-driven co-selection within the soil microbiome.

**Discussion:**

Together, these findings show that long-read genome-resolved metagenomics can uncover how chronic industrial pollution maintains metal-adapted microbial communities while promoting the persistence and potential dissemination of antibiotic resistance in soil ecosystems.

## 1 Introduction

Heavy metal (HM) pollution is a widespread environmental problem affecting ecosystems worldwide (Hou D. et al., 2025; Dagdag et al., 2023). Heavy metals have far-reaching implications for microbial ecology, ecosystem functioning, and public health (Tang et al., 2019; Angon et al., 2024). Unlike organic pollutants, HMs do not degrade and therefore act as long-term selective pressures on environmental microorganisms, fundamentally altering microbial community structure and function (Giller et al., 1998). Industrial activities, particularly mining and metallurgical operations, are primary sources of soil heavy metal pollution, with contaminants including zinc (Zn), lead (Pb), copper (Cu), and cadmium (Cd) accumulating to levels that exceed maximum permissible concentrations in affected regions (Luo et al., 2023). The East Kazakhstan Region exemplifies this challenge, having served as a major industrial center since the 1940s with continuous non-ferrous metal processing activities in cities including Ust-Kamenogorsk and Ridder (Muter et al., 2024; Cherednichenko et al., 2021). These technogenic zones have experienced chronic heavy metal deposition through atmospheric emissions and waste accumulation, creating environments where metal concentrations exceed regulatory limits by multiple orders of magnitude (Woszczyk et al., 2018). The environmental and health consequences of this pollution are substantial, with documented effects on ecosystem stability and increased cancer incidence in affected populations (Rakhimbekova et al., 2024).

Soil microbial communities play essential roles in biogeochemical cycling, organic matter decomposition, and ecosystem resilience. Understanding how these communities respond to chronic heavy metal exposure is therefore critical for assessing ecosystem health and developing effective bioremediation strategies (Zhao et al., 2025; Delgado-Baquerizo et al., 2016). Although heavy metals can exert steong toxic effects on microorganisms, numerous studies have shown that microbial communities often maintain overall diversity under metal stress through compositional shifts that favor metal-resistant taxa (Nikolova et al., 2025; Mi et al., 2025). Recent investigations have further demonstrated that heavy metal contamination disproportionately affects rare taxa and that bacterial community adaptation is driven predominantly by species replacement rather than diversity outright loss (Zhong et al., 2023).

The rapid spread of antibiotic resistance genes (ARGs) poses a serious global health threat. Soil is recognized as one of the major environmental reservoirs of ARGs (Wang F. et al., 2021). Current evidence indicates that heavy metal contamination can drive the co-selection of ARGs together with metal resistance genes (MRGs) within soil microbiomes (Liu et al., 2024). This co-selection arries because resistance genes are frequently co-located on the same mobile genetic elements, including plasmids, transposons, and integrons, which facilitate their simultaneous horizontal transfer (Baker-Austin et al., 2006; Li et al., 2017). The resulting co-selective pressure exerted by heavy metals promotes the dissemination and persistence of antibiotic resistance in environmental reservoirs and can even exert a stronger shaping effect on ARG profiles than direct antibiotic exposure (Shen et al., 2023; Mazhar et al., 2021). Mechanistically, co-resistance occurs when distinct resistance genes are physically linked on the same genetic element, allowing simultaneous selection by either antibiotics or metals (Baker-Austin et al., 2006). Cross-resistance mediated by multidrug efflux pumps and other determinants that protect against both antibiotic and metal stresses (Pal et al., 2017). Co-regulation enables coordinated expression of resistance genes in response to environmental stressors. Together, these processes explain why metal-contaminated soils often harbor elevated abundances of ARGs, even in the absence of direct antibiotic exposure (Wu et al., 2024; Zhang et al., 2024).

The effects of heavy metals on ARG and MRG profiles have been widely investigated, and previous studies have consistently shown that metal contamination can reshape soil resistomes and promote ARG-MRG co-selection (Seiler and Berendonk, 2012; Wang X. et al., 2021). In our previous study, we also found that heavy metal contamination significantly influenced soil taxonomic profiles (Isgandarov et al., 2026). Traditional microbiome characterization approaches, including 16S rRNA gene amplicon sequencing and short-read metagenomics, have provided valuable insights but suffer from limited taxonomic resolution and fragmented genome recovery (Kim N. et al., 2024; Kim C. et al., 2024). Recent advances in long-read sequencing technologies, particularly Oxford Nanopore Technologies (ONT), have revolutionized soil metagenomics by enabling strain-level taxonomic resolution and recovery of complete biosynthetic gene clusters (Bağcı et al., 2025; Burian et al., 2025). Despite the recognized importance of heavy metal-microbiome interactions, several critical knowledge gaps remain (Li et al., 2023; Reznikova et al., 2026). The functional linkages between taxonomic composition, metal resistance mechanisms, and antibiotic resistance profiles have not been comprehensively characterized at the genome-resolved level in chronically contaminated industrial soils (Khrapai et al., 2025).

This study addresses these gaps by applying long-read whole metagenome sequencing to conduct high-resolution taxonomic and functional profiling of soil microbial communities across a gradient of heavy metal contamination in the East Kazakhstan Region. The specific objectives were to: (1) characterize strain-level taxonomic composition and diversity in soils with varying heavy metal concentrations; (2) profile the distribution and abundance of ARGs and MRGs across contaminated sites; (3) evaluate relationships between heavy metal concentrations, soil physicochemical properties, and microbial community structure using multivariate statistical approaches. By leveraging the unprecedented taxonomic resolution afforded by long-read sequencing, this work provides new insights into microbial adaptation strategies in chronically polluted industrial soils and has direct implications for bioremediation and understanding the environmental dimensions of antimicrobial resistance.

## 2 Materials and methods

### 2.1 Sample collection and preparation

#### 2.1.1 Soil sampling area

**Figure 1:**
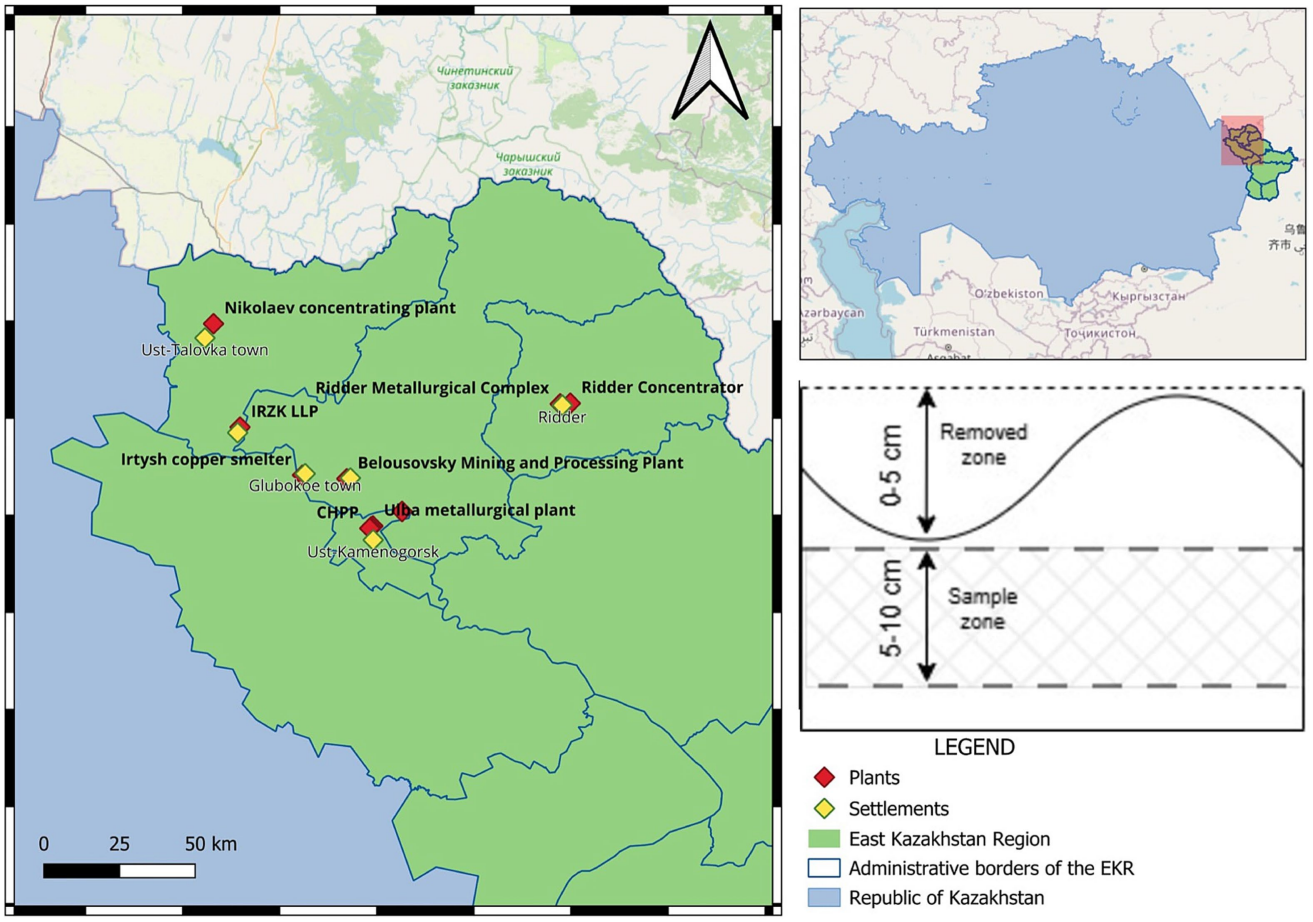
Study area, sampling points, and sampling depth.

Soil samples were collected from the industrial zones of six settlements in the East Kazakhstan Region including Ulba Metallurgical Plant, Ust-Kamenogorsk Metallurgical Complex, and Ust-Kamenogorsk Titanium and Magnesium Plant at Ust-Kamenogorsk city, Irtysh Rare Earth Company at Pervomaisk village, Nikolaevskaya Concentration Plant (Nikolaevsk Concentrator) at Ust-talovka village, Ridder Metallurgical Plant and Ridder Mining and Processing Complex at Ridder city, Belousovka Mining and Processing Plant at Belousovka village, Irtysh Copper Smelter at Glubokoe village (Figure 1). Soil sampling was conducted in the vicinity of copper, zinc and other metal-processing enterprises at East Kazakhstan region. Samples were collected along eight directions corresponding to the wind rose, at distances ranging from 200 to 3,000 m from the boundaries of the industrial sites. The study area is characterized by chestnut, gray-brown, and serozem soils containing varying amounts of rock fragments of different sizes formed under arid conditions. According to data from the national hydrometeorological service of Kazakhstan, the annual precipitation ranges from 200 to 650 mm. The period with an average daily temperature above 0 °C lasts less than 200 days in the north-eastern part of the region The soil samples analyzed in this study were the same as those used in our previous study (Isgandarov et al., 2026). A total of 33 samples were distributed across the settlements as follows: Ust-Kamenogorsk (*n* = 5), TMK (*n* = 4), Pervomaisk (*n* = 6), Shemonaikha (*n* = 5), Ridder (*n* = 5), Belousovka (*n* = 6), and Glubokoe (*n* = 2). Comprehensive details for each sample, including settlement name, site code, GPS coordinates, and physicochemical measurements, are provided in Supplementary Table S1.

#### 2.1.2 Soil sample collection

Thirty-three soil samples were collected during a single field campaign conducted in May 2025 under comparable meteorological conditions. Soils were collected within the zones of industrial influence surrounding the facilities. During soil sampling, each soil sample was divided into subsamples for metagenomic and chemical analyses. Soil for metagenomic analysis was collected at a depth of 5–10 cm in sterile, heat-resistant falcon tubes and immediately placed in liquid nitrogen after collection. The samples were then stored in a freezer at −80 °C. Samples for chemical analysis were collected at a depth of 5–10 cm in approximately 500 g quantities using the envelope method. These samples were placed in cloth bags, air-dried for 1 week at room temperature, and sieved through a 100-mesh sieve.

#### 2.1.3 Heavy metal analysis

Chemical analysis of heavy metals was performed using atomic absorption spectrometry (AAS) with an AA 240 instrument (Agilent, Santa Clara, CA, United States) in accordance with ISO 11047. Soil quality assessment in Kazakhstan is governed by a set of sanitary and hygienic regulations establishing maximum allowable levels of hazardous chemicals in soils. Earlier standards include the hygienic guideline “Maximum Permissible Concentrations of Chemicals in Soil (MPC) No. 3.01.056.97” and SanPiN 42-128-4433-87, which define threshold concentrations of chemical substances to ensure environmental and public health safety. At present, the principal regulatory framework for evaluating soil contamination by inorganic and organic pollutants is the national document “On the Approval of Hygienic Standards for Environmental Safety,” enacted by Order of the Minister of Health of the Republic of Kazakhstan No. RK MH-32 (April 21, 2021). This document establishes legally binding maximum permissible concentrations (MPCs) for priority contaminants in soils and serves as the basis for environmental monitoring and risk assessment.

#### 2.1.4 Soil physiochemical properties evaluation

Soil physicochemical properties were evaluated using standard analytical methods. Soil pH was measured potentiometrically using a pH meter calibrated with three standard buffer solutions (pH 4.01, 6.86, and 9.18) prepared from standard titers. The total instrumental error for pH measurement did not exceed 0.1 pH units. Soil moisture content (H₂O) was determined gravimetrically by drying soil samples to constant weight at 105 °C.

Easily hydrolysable nitrogen (N₂) was determined according to the Tyurin–Kononova method. Briefly, soil samples were extracted with 0.5 N H₂SO₄ and subjected to 24 h hydrolysis. Ammonia was then distilled into a boric acid solution after addition of an alkali, and the distillate was quantified by titration with a standard acid.

Mobile forms of phosphorus (P₂O₅) and potassium (K₂O) in calcareous soils were extracted and analyzed using the Machigin method in accordance with GOST 46-42-76. Extractions were performed using a 1% ammonium carbonate ([NH₄]₂CO₃) solution adjusted to pH 9.0. The concentration of P₂O₅ was determined colorimetrically as a blue phosphorus–molybdenum complex using a photoelectric colorimeter, and mobile K₂O was quantified using a flame photometer.

### 2.2 DNA extraction, library construction, and sequencing

Genomic DNA was isolated from the 33 selected soil samples individually as a single unit using the DNeasy PowerSoil Kit (Qiagen, Germany) in strict accordance with the manufacturer’s protocol. No technical or biological replicates were generated. The extracted DNA was subsequently cleaned and size-selected with AMPure XP magnetic beads (Beckman Coulter, United States) to achieve the required purity and concentration. DNA quality was evaluated spectrophotometrically with a NanoDrop 2000 instrument (Thermo Fisher Scientific, United States) by assessing A₂₆₀/₂₈₀ and A₂₆₀/₂₃₀ absorbance ratios.

Sequencing libraries were constructed using the Native Barcoding Kit 24 V14 (SQK-NBD114.24; Oxford Nanopore Technologies, UK). Prepared libraries were loaded onto R10.4.1 flow cells and sequenced on a PromethION 2 Solo platform (Oxford Nanopore Technologies) under the control of MinKNOW software (v. 25.05.14). Signal processing, including basecalling and barcode demultiplexing, was carried out within the MinKNOW environment using parameters corresponding to the applied chemistry and library kit.

### 2.3 Metagenomic analysis

#### 2.3.1 Quality control

After sequencing, raw reads were analyzed and processed using the NanoPack toolkit. Read quality filtering was performed with NanoFilt (version 2.8.0), and NanoPlot (version 1.46.2) was used to visualize read length and quality distributions. Reads were filtered using a minimum quality score threshold of Q10 and minimal read length of 300 bp.

#### 2.3.2 Taxonomic annotation

Taxonomic mapping and classification of filtered reads were performed using MetaMaps (Version 0.1.633d2e0-0) with default parameters. MetaMaps was selected because it provides accurate taxonomic annotation of long-read sequences and supports strain-level resolution (Dilthey et al., 2019). Reads were mapped against a RefSeq database with functional annotations.^1^ After taxonomic classification, unmapped reads and reads assigned to viruses, archaea, and eukaryotes were removed from downstream analyses.

#### 2.3.3 Profiling of ARGs and MRGs

The abundance and diversity of ARGs were profiled using the DeepARG tool (version 1.0.4). DeepARG utilize deep learning architecture for ARG identification (Arango-Argoty et al., 2018). Nucleotide sequences were annotated against the DeepARG database utilizing the long sequence (LS) model. To ensure high-confidence ARG predictions and minimize false positives, strict prediction parameters were applied: a minimum probability cutoff of 0.8, a minimum alignment identity of 50%, and an *E*-value < 1 × 10^−5^.

MRGs were identified by aligning quality-trimmed reads against the experimentally confirmed BacMet database (version 2.0, accessed on 15 February 2026) using the DIAMOND sequence aligner (version 2.1.22) in *blastx* mode (Buchfink et al., 2021). To eliminate potential false positives, raw alignment hits were filtered using the following thresholds: percentage identity > 60%, alignment length > 30 amino acids, and E-value < 1 × 10^−5^.

#### 2.3.4 Functional profiling

The functional profiles of the soil microbial communities were evaluated using the MetaMaps pipeline against the curated RefSeq database augmented with gene location and functional annotation data. Following the initial alignment, the metamaps classify module was used to assign reads to specific genomic features. Based on these assignments, Clusters of Orthologous Groups (COG) and KEGG orthology profiles were generated to characterize the functional potential of the communities.

#### 2.3.5 Downstream and statistical analysis

All downstream bioinformatic processing, ecological calculations, and statistical visualizations were performed in the R environment (version 4.5.1). Alpha diversity indices, including Shannon, Simpson, Chao1, ACE, and observed species/gene Richness, were calculated for taxonomic (OTU), antibiotic resistance gene (ARG), and metal resistance gene (MRG) profiles using the vegan package (version 2.7-2). To account for variations in sequencing depth prior to multivariate analysis, the raw abundance count matrices were subjected to Hellinger transformation using the decostand function in vegan. For beta diversity, Bray–Curtis dissimilarity matrices were constructed from the Hellinger-transformed taxonomic and functional profiles. The variance in community structure explained by heavy metal gradients was quantified using Permutational Multivariate Analysis of Variance (PERMANOVA) via the adonis2 function (999 permutations). To visualize community structure shifts along environmental gradients, Non-metric Multidimensional Scaling (NMDS) were generated based on Bray-Curtis dissimilarities. The relationships between community structures (taxa, ARGs, MRGs) and continuous environmental variables (Zn, Pb, Cu, Cd, P^2^O^5^, pH, K_2_O, N_2,_ H_2_O) were further evaluated using Redundancy Analysis (RDA). Global and term-specific significance of the RDA models were assessed via permutation tests for Redundancy Analysis. Spearman’s rank correlation coefficients were calculated using the Hmisc package (version 5.2-5) to identify significant bivariate associations between the relative abundances of the top 20 dominant taxa/genes, alpha diversity metrics, and soil physicochemical properties. Correlation matrices were visualized as hierarchical clustered heatmaps using the pheatmap package, with statistical significance seted as (*p* < 0.05).

#### 2.3.6 Network construction

To comprehensively investigate microbial community dynamics and the potential for heavy metal-driven co-selection of antibiotic resistance, three distinct networks were constructed and analyzed. Tripartite Genomic Association Network constructed to map direct physical linkages, a tripartite association network was constructed connecting microbial strains to their respective MRGs and ARGs based on genomic colocalization. Primary Co-occurrence Network constructed to evaluate broader ecological co-occurrence patterns, a primary statistical co-occurrence network integrating microbial strains, MRGs, and ARGs was constructed. This network was generated based on robust Spearman’s rank correlation matrices to ensure the biological relevance of the associations. Bipartite ARG-MRG Co-occurrence Subnetwork constructed to explicitly investigate statistical co-selection dynamics between the two resistance classes, a targeted bipartite co-occurrence network was constructed modeling correlations exclusively between ARGs and MRGs. Across all three networks, isolated nodes were removed prior to downstream analysis to focus strictly on the interactive core community and resistome. To evaluate structural connectivity and functional compartmentalization, global topological properties were calculated across the networks, including node degree, betweenness centrality, clustering coefficient, and modularity. To validate that the statistical co-occurrence networks were driven by deterministic biological processes rather than random chance, the empirical networks were compared against Erdős-Rényi random null models. For each empirical network, 100 random networks with identical node and edge counts were generated. The clustering coefficient and modularity of the empirical networks were compared to the mean and standard deviation of their corresponding random networks, with statistical significance determined via Z-tests (*p* < 0.001). Additionally, degree distributions were analyzed using goodness-of-fit tests to verify that the networks exhibited scale-free topology, characteristic of biological power-law distributions (*p* > 0.05 indicating a scale-free structure). To identify keystone taxa driving community assembly and network stability within the primary co-occurrence network, nodes were classified based on their topological roles within and between network modules. This classification utilized within-module connectivity (Zi) and among-module connectivity (Pi), categorizing nodes into four ecological roles based on established thresholds: Peripheral nodes (Zi ≤ 2.5, Pi ≤ 0.625): Nodes with few links, mostly localized within their own modules. Connectors (Zi ≤ 2.5, Pi > 0.625): Nodes highly linked to several different modules, mediating broad network interactions. Module Hubs (Zi > 2.5, Pi ≤ 0.625): Highly connected nodes functioning as central organizers within their respective modules. Network Hubs (Zi > 2.5, Pi > 0.625): Highly connected nodes serving as global hubs across multiple modules. Furthermore, basic node degree centralities were evaluated across all networks to identify “super-hosts” (highly connected taxa) and “hub genes” (widely disseminated ARGs and MRGs) that structurally anchor the resistome. All statistical analyses, network construction, and null model generation were performed using R with igraph package (version 2.2.2). Final network visualization and spatialization were conducted using Gephi (version 0.10.1).

## 3 Results

### 3.1 Soil properties and heavy metal concentration

The physicochemical properties and heavy metal concentrations of the sampled soils were evaluated and are summarized in Supplementary Tables S1, S2. The pH of the sampled soils ranged from 5.49 to 7.42, with an average of 6.46 (± 0.58), indicating that the soil environments are predominantly slightly acidic to near-neutral. Available soil macronutrients exhibited considerable variation across the sampling sites. Nitrogen (N₂) levels ranged from 30.80 to 187.60 mg/kg (mean: 72.97 ± 38.23 mg/kg), while available phosphorus (P₂O₅) ranged from 6.00 to 288.00 mg/kg (mean: 80.12 ± 73.03 mg/kg). Potassium (K₂O) was generally abundant, ranging from 160.00 to 1080.00 mg/kg (mean: 491.82 ± 239.24 mg/kg). Soil moisture content (H₂O) remained relatively low across all sites, ranging from 0.48 to 4.90% (mean: 2.11 ± 0.95%). Analysis of heavy metal concentrations revealed extreme variability and significant point-source contamination across the different sampling locations (*p* < 0.05). Zinc (Zn) was the most abundant heavy metal, with concentrations ranging drastically from 1.50 to 3624.00 mg/kg, yielding a highly variable mean of 463.16 mg/kg (± 883.41 mg/kg; CV = 190.73%). Copper (Cu) levels ranged from 0.50 to 762.00 mg/kg (mean: 48.91 ± 154.55 mg/kg), with the highest concentration observed at the Glubokoe (G1) site. Similarly, cadmium (Cd) and lead (Pb) concentrations exhibited strong positive skewness due to localized contamination spikes. Cadmium levels ranged from 0.40 to 118.00 mg/kg (mean: 10.82 ± 22.51 mg/kg), and lead concentrations ranged from 0.60 to 227.00 mg/kg (mean: 19.53 ± 43.16 mg/kg). Notably, the highest levels of metal contamination were consistently recorded in the Ridder (R2, R3, R5) and Ust-Kamenogorsk (U2, U3, U4) sampling sites, indicating severe, localized heavy metal stress within these specific environmental gradients.

### 3.2 Taxonomic abundance

**Figure 2:**
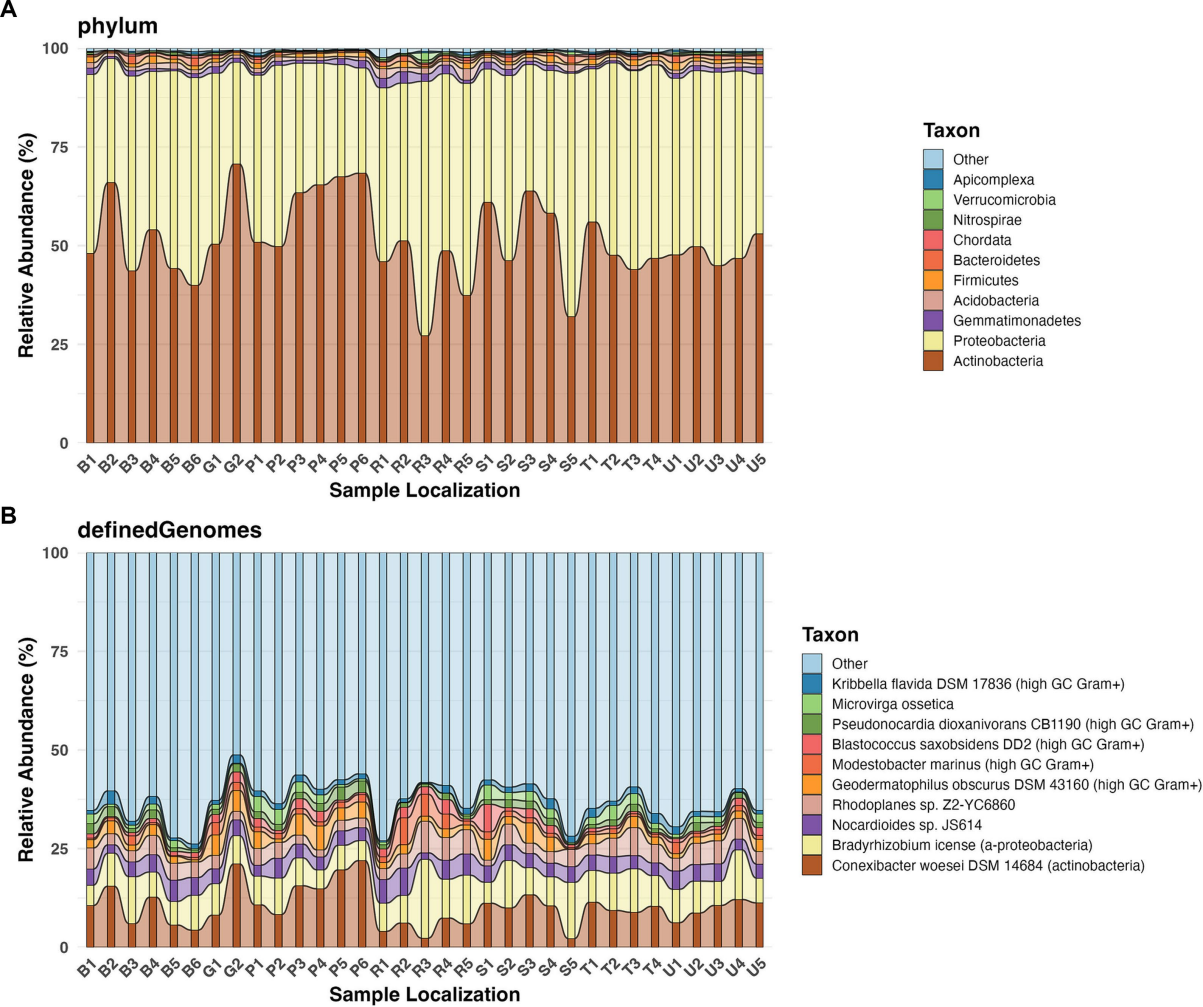
Relative abundance of top 10 taxa: **(A)** Relative abundance at phylum level; **(B)** Relative abundance at strain level.

Metagenomic analysis across all soil samples identified a highly diverse microbial community comprising 37 distinct phyla, 165 orders, 390 families, 1,037 genera, 2,697 species, and 3,053 strains. The community was co-dominated by two phyla: Actinobacteria (51.2%) and Proteobacteria (42.9%), which together accounted for 94.1% of the total relative abundance (Figure 2A). Minor phyla included Gemmatimonadetes (1.18%), Acidobacteria (1.13%), and Firmicutes (0.99%). High-resolution taxonomic profiling at the strain level revealed that *Conexibacter woesei* DSM 14684 was the most abundant strain, constituting 10.2% of the total community, followed closely by *Bradyrhizobium icense* at 8.52%. *Nocardioides* sp. JS614 and *Rhodoplanes* sp. Z2-YC6860 each contributed 4.00% to the overall abundance, while *Geodermatophilus obscurus* DSM 43160 represented 2.68% (Figure 2B). The strains ranked 6th through 10th in abundance - *Modestobacter marinus, Blastococcus saxobsidens DD2, Pseudonocardia dioxanivorans CB1190, Microvirga ossetica*, and *Kribbella flavida DSM 17836* — each accounted for 1.47–1.61% of total abundance.

**Figure 3:**
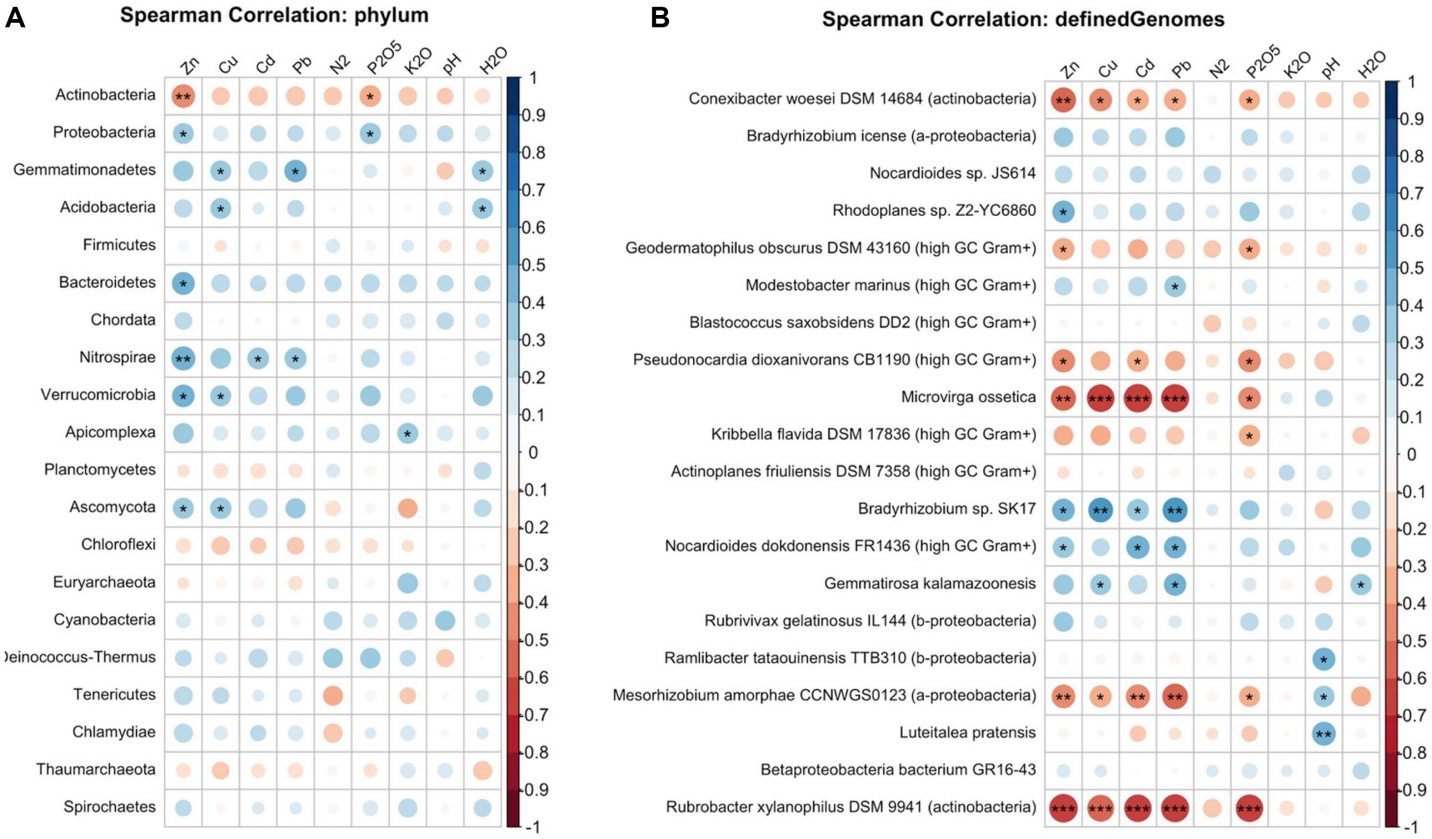
Spearman correlations of soil physiochemical properties and heavy metals with microorganisms at **(A)** phylum level; **(B)** at strain level. Statistical significance is denoted by asterisks: *p < 0.05, **p < 0.01, ***p < 0.001.

Spearman rank correlation analysis revealed significant associations between soil physicochemical variables and microbial composition (Figure 3). At the phylum level, Actinobacteria exhibited significant negative correlations with Zn and P₂O₅ (Figure 3A). In contrast, Proteobacteria demonstrated significant positive associations with both Zn and P₂O₅. Gemmatimonadetes showed significant positive correlations with H₂O, Cu, and Pb. Nitrospirae was positively correlated with Cd, Pb, and Zn. Acidobacteria correlated positively with Cu and H₂O. Ascomycota showed positive associations with Cu and Zn. Furthermore, Bacteroidetes exhibited a strong positive correlation specifically with Zn. t the strain level, distinct response patterns to heavy metal concentrations and soil properties were observed (Figure 3B). Strains such as *Rubrobacter xylanophilus* DSM 9941, *Microvirga ossetica*, and *Conexibacter woesei* DSM 14684 exhibited strong negative correlations with Cu, Cd, Pb, Zn, and P₂O₅. Similarly, *Mesorhizobium amorphae* CCNWGS0123 displayed significant negative correlations with all measured heavy metals and P₂O₅, while uniquely serving as the only strain to show a significant positive correlation with soil pH. Conversely, several strains demonstrated high tolerance to environmental contamination. *Bradyrhizobium* sp. SK17 exhibited significant positive correlations with all measured heavy metals. Additionally, *Gemmatirosa kalamazoonesis*, *Nocardioides dokdonensis* FR1436, *Rhodoplanes* sp. Z2-YC6860, and *Modestobacter marinus* were positively associated with multiple heavy metals, suggesting robust adaptation mechanisms to localized metal stress.

### 3.3 Abundance of ARGs and MRGs

**Figure 4:**
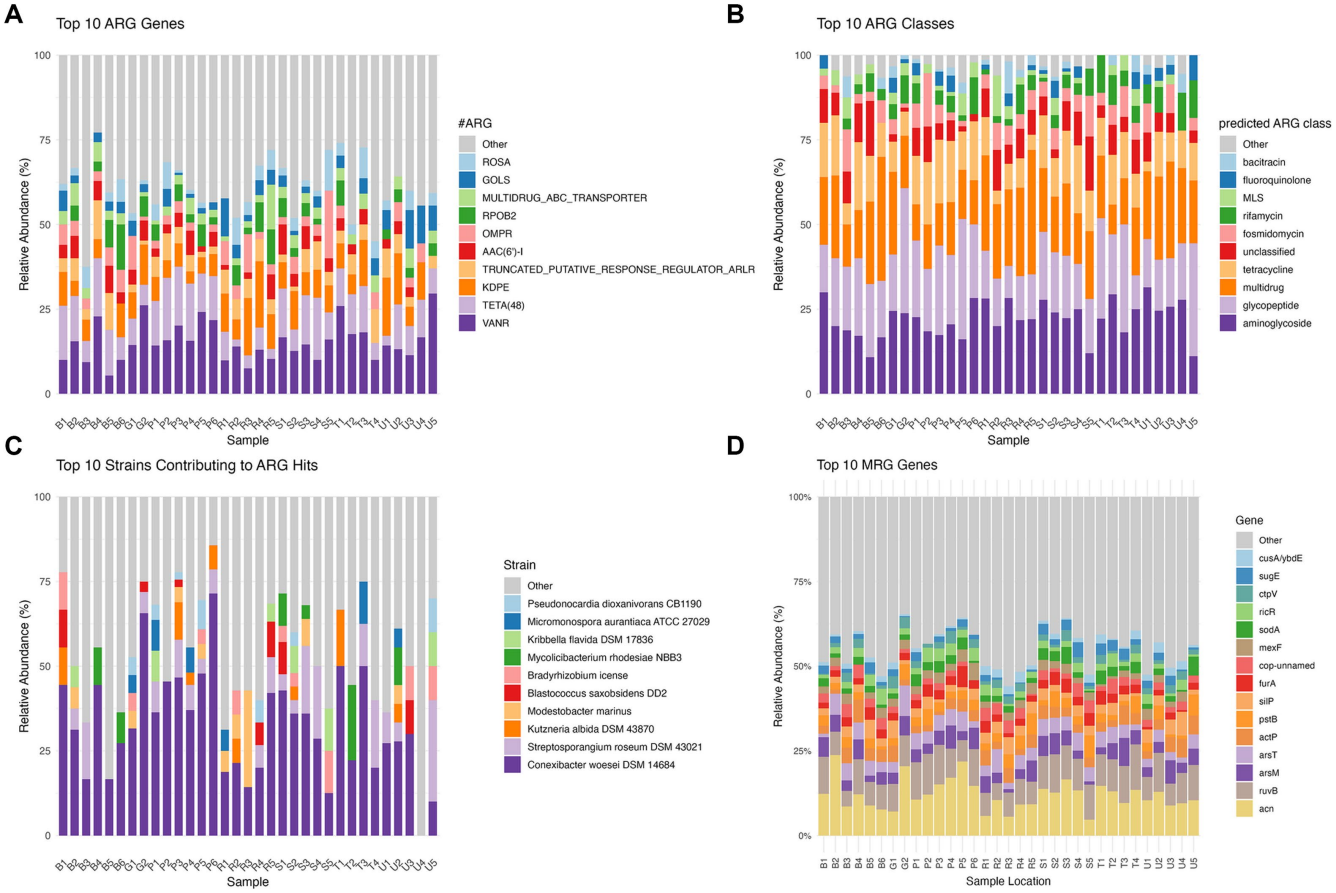
Relative abundance stacked bar chart of top **(A)** ARGs; **(B)** ARG classes; **(C)** Strains contributing to ARGs; **(D)** MRGs.

Metagenomic profiling revealed a highly diverse resistance genes across the sampled soils, identifying ARGs associated with 18 distinct drug classes. Aminoglycoside resistance genes were the most prevalent, accounting for 22.5% of the total assigned ARGs (Figure 4A). This was followed closely by glycopeptide and multidrug resistance classes, which represented 20.0 and 19.4% of the total relative abundance, respectively. At the individual gene level, 125 distinct ARGs were identified. The most highly enriched gene was *vanR*, a regulator of glycopeptide resistance, which accounted for 15.5% of the total ARG abundance (Figure 4B). The tetracycline efflux pump gene *tetA*(48) was the second most abundant (11.1%), followed by the two-component sensor kinase gene *kdpE* (8.39%). Analysis of the taxonomic hosts associated with these ARGs revealed that the soil resistome was predominantly concentrated within specific bacterial strains. *Conexibacter woesei* DSM 14684 was identified as the primary ARG host, harboring 36.5% of the total assigned ARGs. *Streptosporangium roseum* DSM 43021 was the second most prominent host, associated with 6.97% of the total ARGs (Figure 4C). Spearman rank correlation analysis revealed complex co-occurrence patterns among these resistance determinants and environmental drivers. Notably, the *ompR* and *bacA* genes exhibited statistically significant positive correlations with several heavy metals (Zn, Cu, Cd, and Pb) (Supplementary Figure S1).

Furthermore, the soils harbored an extensive suite of metal resistance genes (MRGs), with a total of 238 distinct MRGs identified across all samples. The most abundant MRG detected was *czcA* (confirm gene name, previously listed as “can”), followed by *ruvB*, *arsM*, and *arsT* (Figure 4D). Spearman correlation analysis demonstrated highly significant internal co-occurrences among the MRGs themselves, as well as strong associations with the soil physicochemical gradient. Specifically, the metal efflux and tolerance genes *silP*, *nczA*, *cusA/ybdE*, *ctpG*, *arsB*, and *copB* were significantly and positively correlated with soil heavy metal concentrations (Zn, Cu, Cd, and Pb) (Supplementary Figure S2), indicating that heavy metal contamination exerts strong selective pressure enriching for these specific genetic determinants.

### 3.4 Alpha diversity of microorganisms, ARGs, MRGs

The alpha diversity of the taxonomic community, ARGs, and MRGs was evaluated using Shannon, Simpson, Chao1, ACE, and Richness indices (Supplementary Table S2). Taxonomic alpha diversity metrics indicated an average Richness of 1,386 (range: 865–1864) and an average Shannon diversity index of 5.09. ARG profiles exhibited an average Richness of 24 (range: 12–42) and an average Shannon index of 2.84. For MRGs, the average Richness was 76.6 (range: 44–106) with a mean Shannon index of 3.74.

**Figure 5:**
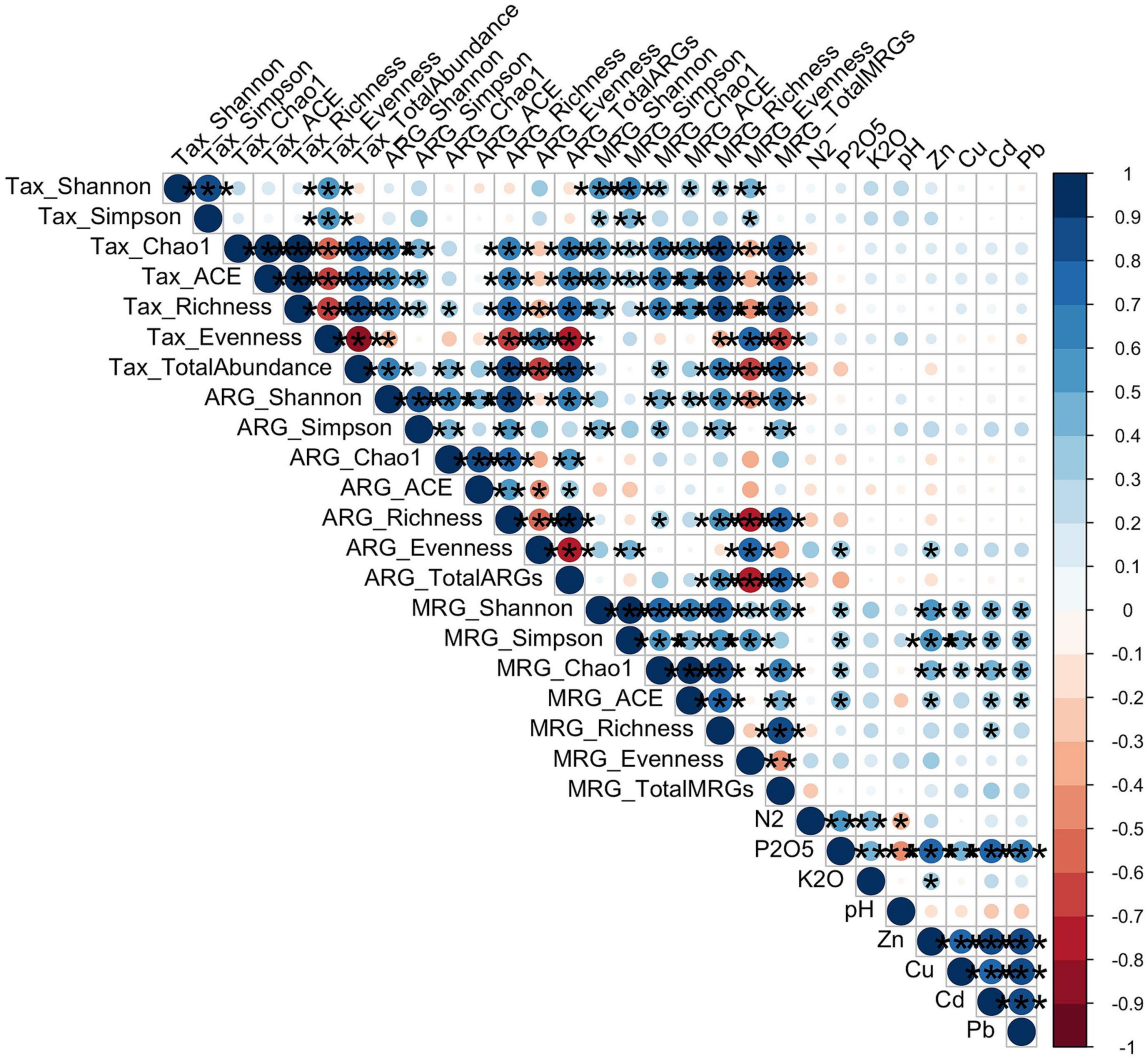
Spearman correlation of soil physiochemical properties, heavy metals, and alpha diversity indices. Statistical significance is denoted by asterisks: *p < 0.05, **p < 0.01, ***p < 0.001.

Spearman correlation analysis was conducted to identify the relationships among the alpha diversity metrics of the three components (Taxonomy, ARGs, MRGs) and soil physicochemical properties (Figure 5). Strong positive correlations were observed between several taxonomic diversity indices and both ARG and MRG diversity. Taxonomic Shannon and Simpson indices were positively correlated with MRG Shannon, Simpson, Chao1, ACE, and Richness indices (all *p* < 0.01). Furthermore, taxonomic estimators (Chao1, ACE, and Richness) displayed highly significant positive associations with ARG Chao1, ACE, and Richness, as well as MRG Shannon, Simpson, Chao1, ACE, and Richness (*p* < 0.05). Additionally, ARG alpha diversity (Shannon and Simpson) exhibited strong positive correlations with MRG Shannon, Simpson, Chao1, ACE, and Richness (*p* < 0.05).

Environmental factors demonstrated distinct impacts on the alpha diversity metrics. Soil heavy metals, exhibited significant positive correlations with both MRG Shannon, Simpson and Chao1 indices (*p* < 0.05), indicating that higher concentrations of these metals were associated with higher MRG diversity. Among macronutrients, P2O5 showed highly significant positive correlations with multiple MRG indices (*p* < 0.05).

### 3.5 Beta diversity

Beta diversity applied on bray-curtis distance matrix of Taxonomic communities, ARGs and MRGs. PERMANOVA confirmed that heavy metals significantly explained variation in MRG, ARG, and strain-level community composition. For MRGs, the combined effect of Zn, Cu, Pb, and Cd accounted for 17.8% of the total variation (*R*^2^ = 0.1781, *F* = 1.52, *p* = 0.003). For ARG profiles, the same set of metals explained 15.9% of the variation (*R*^2^ = 0.1588, *F* = 1.32, *p* = 0.033). At the strain level, the model including localization and the four metals together explained 40.3% of the compositional variation (*R*^2^ = 0.4028, *F* = 1.48, *p* = 0.013). These results indicate that both heavy metal contamination and spatial localization jointly shape the structure of MRG, ARG, and microbial strain communities across the studied soils. Non-metric multidimensional scaling (NMDS, Bray–Curtis) utilized for visualization. NMDS revealed clear structuring of MRG, ARG, phylum and strain-level communities along heavy metal gradients (Supplementary Figure S3). At all ordinations stress levels were acceptable (stress < 0.2).

RDA general model revealed that heavy metals significantly shaped the distribution of MRGs (*p* = 0.004), ARGs (*p* = 0.046), and microbial strains (*p* = 0.003) across the sampled sites (Supplementary Figure S4). Across all global RDA models, Zinc (Zn) emerged as the most dominant independent driver of community and resistome variation (*p* = 0.001), followed closely by soil pH (*p* = 0.006). The RDA biplots demonstrated strong collinearity among the vectors for Zn, Pb, Cd, Cu, H₂O, and P₂O₅ along the primary axis, reflecting a robust, localized co-occurrence of these heavy metals and soil parameters. Taxon-specific responses were clearly delineated along this contamination gradient. Highly tolerant strains, including *Bradyrhizobium icense*, *Bradyrhizobium* sp. 385S1 MB, and *Modestobacter marinus*, exhibited direct, positive associations with the heavy metal vectors. In stark contrast, *Conexibacter woesei* DSM 14684, *Rubrobacter xylanophilus* DSM 9941, *Microvirga ossetica*, and *Mycobacterium* sp. JS623 plotted in the exact opposite direction, confirming their acute sensitivity to elevated heavy metal loads. Notably, the directional alignment of multiple ARGs and specific MRGs with the heavy metal vectors highlights a distinct pattern of co-selection, demonstrating that persistent heavy metal pressure actively enriches for dual-resistance mechanisms within the soil resistome.

### 3.6 Functional annotation

**Figure 6:**
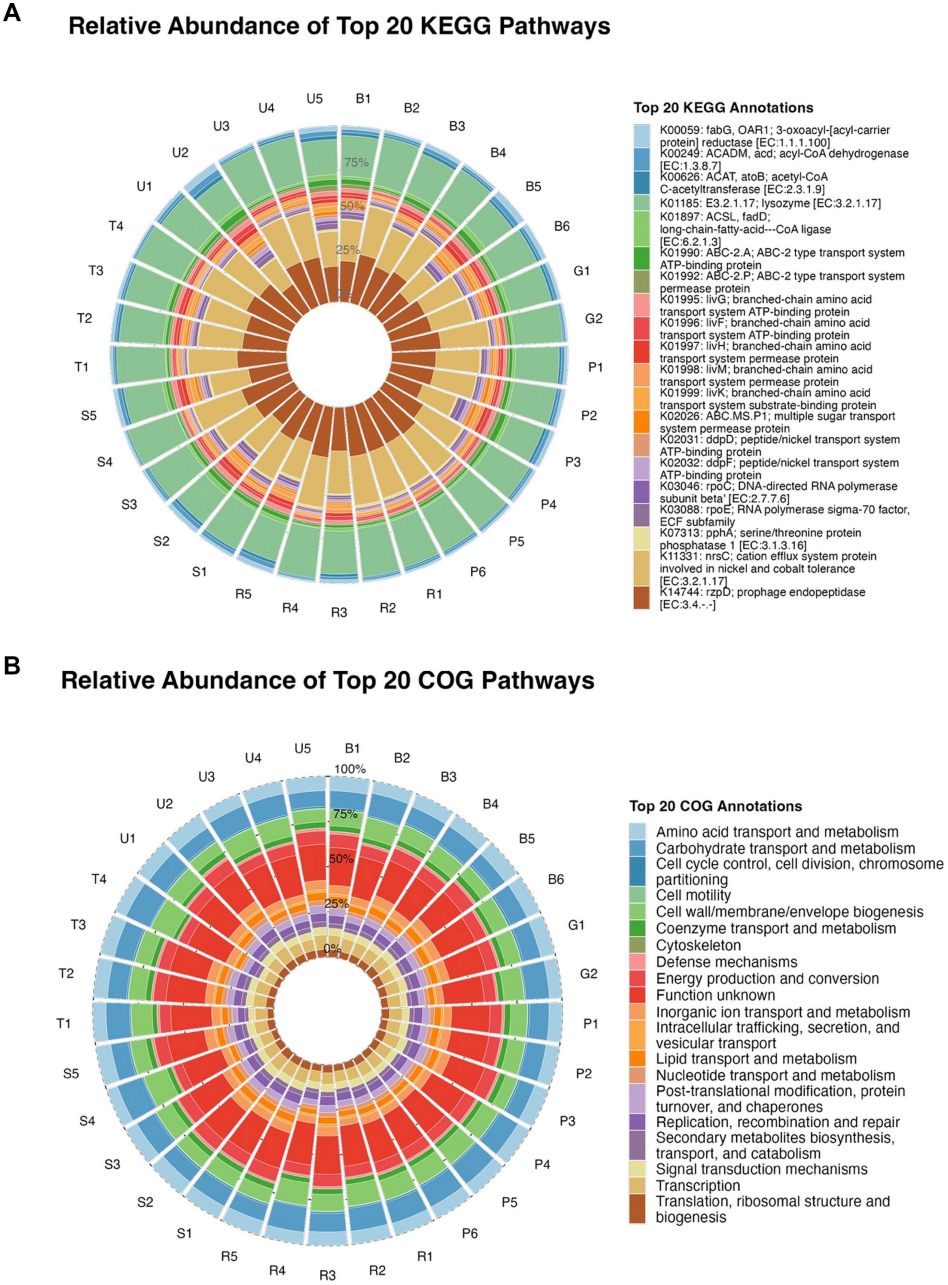
Circular plot of top 20 annotated **(A)** KEGG pathways across all samples; **(B)** COG pathways across all samples.

Functional annotation of data through KEGG database revealed several important metabolic functions in microbial communities. Functional annotation against the KEGG database showed that the top 20 KEGG orthologs were dominated by transport systems and energy-related enzymes (Figure 6A). Abundant functions included multiple ABC-2 type transport system permease and ATP-binding proteins (K01990, K01991, K01992), branched-chain amino acid transport components (e.g., K01995, K01996, K01997, K01998, K01999), and peptide/nickel transport systems (K02031, K02032). Key metabolic enzymes such as acyl-CoA dehydrogenase (K00249), acetyl-CoA C-acetyltransferase (K00626), long-chain-fatty-acid—CoA ligase (K01897), and RNA polymerase sigma factor and associated regulatory proteins (K03088, K03089, K07313, K11331) were also consistently represented across samples.

Correlation analysis between KEGG ortholog abundances and heavy metals indicated strong negative associations for several transport- and transcription-related genes (Supplementary Figure S5A). Multiple ABC-2 type transporters (K01990, K01991, K01992) and branched-chain amino acid transport proteins (K01995–K01999) showed significant negative correlations with Zn and Cd (Zn: *p* < 0.05–0.01; Cd: *p* < 0.01), suggesting suppression of these transporter functions under higher metal loads. Similarly, genes encoding peptide/nickel transport systems (K02031, K02032) and DNA-directed RNA polymerase subunits and sigma factors (K03088, K03089, K07313, K11331) were negatively correlated with Zn, Pb, and Cd (all *p* < 0.01), indicating broad functional constraints on transport and transcription in heavily contaminated soils. In contrast, only a few KEGG orthologs displayed weak positive correlations with individual metals, and none reached the same level of significance as the negatively associated transport and transcription functions.

Across all samples, the functional potential inferred from COG annotations was dominated by pathways related to core cellular processes (Figure 6B). The most abundant categories were defense mechanisms, energy production and conversion, and amino acid, carbohydrate, and coenzyme transport and metabolism, together accounting for the majority of the predicted functional repertoire. Functions associated with cell wall/membrane/envelope biogenesis, cell motility, inorganic ion transport and metabolism, and signal transduction mechanisms were also consistently represented across sites, whereas categories such as cytoskeleton, transcription, and translation, ribosomal structure and biogenesis contributed relatively smaller proportions of the functional profile.

Spearman correlation analysis revealed distinct relationships between COG category abundances and heavy metal concentrations (Supplementary Figure S5B). Cytoskeleton-related functions showed significant negative correlations with both Cu and Cd (Cu: *p* < 0.05; Cd: *p* < 0.01), indicating reduced representation of these pathways in highly contaminated soils. In contrast, inorganic ion transport and metabolism exhibited a significant positive correlation with Zn (p < 0.05), suggesting enrichment of ion-transport functions along the Zn gradient. Additionally, signal transduction mechanisms were positively correlated with Zn (*p* < 0.05) but negatively associated with Cd (*p* < 0.05), highlighting metal-specific functional shifts in regulatory pathways.

### 3.7 Network analysis

**Figure 7:**
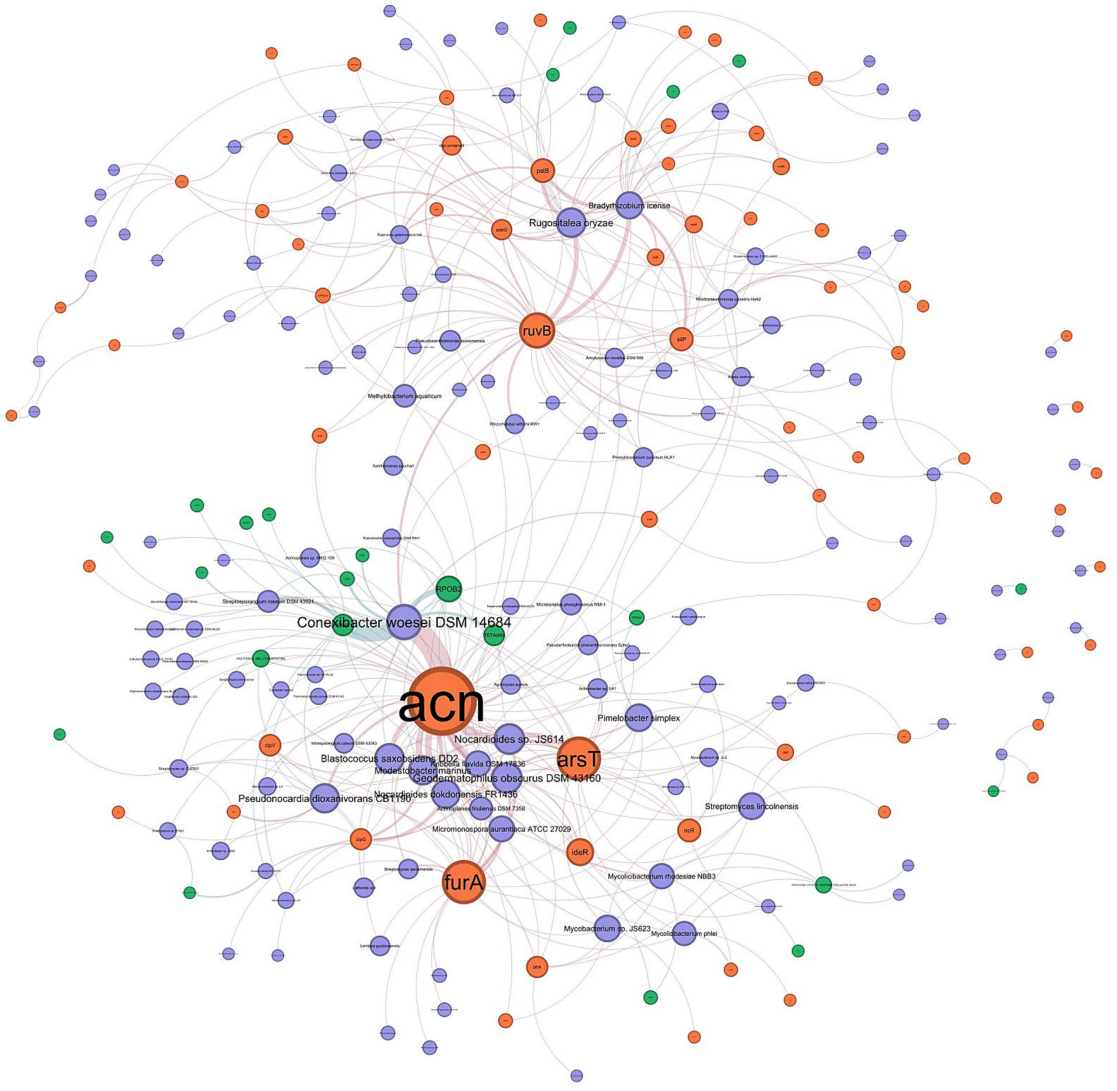
Tripartite network with direct matches of strains, ARGs, and MRGs.

**Figure 8:**
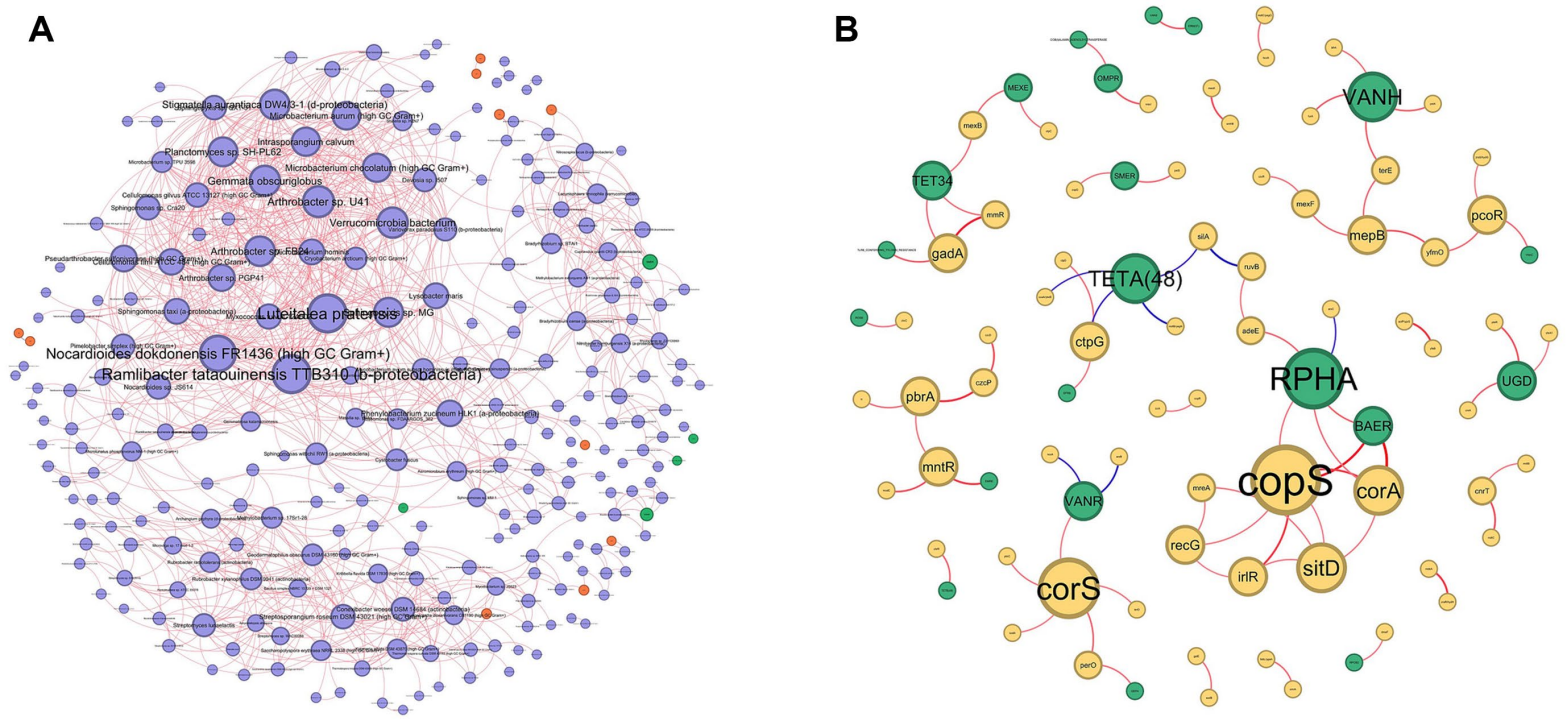
Sperman correlation based co-occurrence network of **(A)** Microbial communities with ARGs and MRGs; **(B)** Co-occurrence ARGs with MRGs.

To investigate the potential for heavy metal-driven co-selection of antibiotic resistance, a tripartite association network was constructed mapping direct genomic linkages among microbial strains, MRGs, and ARGs. To focus exclusively on the core resistome architecture, isolated nodes were excluded from the analysis. The resulting robust network comprised 231 core nodes interconnected by 418 edges. Within this network space, specific microbial taxa emerged as vital ecological reservoirs and structural bridges linking diverse resistance determinants. Consistent with taxonomic abundance profiles, *Bradyrhizobium icense* and *Conexibacter woesei* DSM 14684 exhibited the highest degree centralities (degrees = 23 and 18, respectively). Along with *Rugositalea oryzae* (degree = 16), these taxa functioned as primary “super-hosts,” structurally anchoring the co-selection network and driving the mobilization of resistance. The metal resistome component of the network was overwhelmingly dominated by the *acn* and *ruvB* genes, which demonstrated massive cross-taxa distribution and extreme network centrality (degrees = 52 and 32, respectively). Along with *furA* (degree = 26), these genes constitute the core heavy metal resistance hubs within the microbial community. Concurrently, within the antibiotic resistome, glycopeptide (*VANR*) and rifamycin (*RPOB2*) resistance determinants were the most widely disseminated ARGs, exhibiting the highest degree centralities among antibiotic resistance nodes (degrees = 11 and 10, respectively). The tetracycline resistance gene *tetA(48)* also displayed high connectivity (degree = 6). The pronounced topological prominence of these specific ARGs and MRGs within shared microbial hosts strongly supports the occurrence of linked co-selection under environmental heavy metal stress (Figures 7, 8).

To evaluate significant co-occurrence patterns among microbial strains, MRGs, and ARGs, a primary co-occurrence network was constructed based on robust correlations (*r* > 0.7, *p* < 0.01). The total network comprised 357 nodes (242 strains, 5 ARGs, and 10 MRGs) and 1,084 edges. Topological analysis confirmed that the network followed a scale-free, biological power-law distribution (*p* > 0.05). Furthermore, analysis revealed significant structural divergence between the observed microbial co-occurrence network and Erdős-Rényi random null models. The empirical network exhibited a significantly higher clustering coefficient (Real: 0.5245 vs. Random: 0.0329 ± 0.0031; *Z* = 156.32, *p* < 0.001) and modularity (Real: 0.6043 vs. Random: 0.3132 ± 0.0061; *Z* = 47.48, *p* < 0.001). This confirms a non-random, highly connected topological structure indicative of intense biological co-selection and modular niche partitioning under heavy metal stress. Within the community structure, specific nodes played disproportionate roles in network topology. *Luteitalea pratensis*, *Ramlibacter tataouinensis* TTB310, *Nocardioides dokdonensis* FR1436, *Arthrobacter* sp. U41, and *Gemmata obscuriglobus* were identified as high-degree taxa, highlighting their role in the regulation of the community. Among resistance genes, *smeR* and *vanH* emerged as hub ARGs with node degrees of 9 and 6, respectively.

To further delineate keystone taxa driving community assembly, nodes were categorized based on their within-module connectivity (*Zi*) and among-module connectivity (*Pi*) (Supplementary Figure S6). *Conexibacter woesei* DSM 14684, *Streptosporangium roseum* DSM 43021, and *Phenylobacterium zucineum* HLK1 were identified as Module Hubs (*Zi* > 2.5), acting as central organizers within their respective functional sub-communities. Concurrently, *Frankia alni* ACN14a and *Pseudarthrobacter phenanthrenivorans* Sphe3 functioned as Connectors (*Pi* > 0.625), mediating interactions across different modules. Notably, the majority of these keystone species belong to Actinobacteria, underscoring their vital ecological role in network stability and structural cohesion.

To specifically evaluate ARG-MRG co-selection dynamics, a secondary co-occurrence network was constructed based on significant correlations between resistance genes (*r* > 0.5, *p* < 0.01). This targeted bipartite network encompassed 88 nodes (21 ARGs and 67 MRGs) and 76 edges. Similar to the broader community network, this ARG-MRG co-occurrence network demonstrated a scale-free topology (*p* > 0.05) and exhibited structural parameters significantly distinct from random models, featuring elevated clustering (*Z* = 11.01, *p* < 0.001) and modularity (*Z* = 4.74, *p* < 0.001). Within this network, the MRGs *copS*, *corS*, and *corA* exhibited the highest node degrees (6, 5, and 4, respectively), establishing their roles as primary hub genes mediating ARG-MRG interactions. Concurrently, the ARGs *rphA*, *vanH*, and *tetA(48)* demonstrated peak connectivity with degrees of 5, 4, and 4, respectively. The abundance of both positive and negative significant correlations between these gene classes highlights the strong potential for linked ARG-MRG co-selection mechanisms within these environmental microbial communities.

## 4 Discussion

This Srudy provides genome-resolved metagenomic view of chronic heavy metal-polluted soils using ONT long-read sequencing technology. Our study revealed strain-level microbial communities and direct evidence of heavy metal-driven co-selection of antibiotic and metal resistant genes. Long-read sequencing identified 3,053 distinct strains acroos all sample and show high diversity communities under chronic heavy metal pollution effect. The Dominance of Actinobacteria and Proteobacteria aligns with recent findings from chronically contaminated mining sites (Mi et al., 2025; Nikolova et al., 2025). These phyla could be predominated due to their metabolic versatility and intrinsic metal tolerance mechanisms (Zhu et al., 2025; Li et al., 2024). In contrast, our analyses revealed statistically significant decreasing of relative abundance of Actinobacteria under high Zn concentrations and P_2_O_5_. The suppression of Actinobacteria under extreme Zn stress is likely driven by the disruption of intracellular metal homeostasis. High Zn concentrations competitively inhibit the cellular uptake of essential trace metals like manganese and iron, leading to severe oxidative stress and imposing a high metabolic cost for maintaining heavy metal efflux systems (Xia et al., 2021; Lonergan and Skaar, 2019; Raghavan et al., 2023). Furthermore, the negative correlation with P_2_O_5_ can be attributed to competitive exclusion. Actinobacteria often thrive in oligotrophic environments by utilizing their robust phosphate-solubilizing capabilities (Suriyachadkun et al., 2022; Wang et al., 2022). However, in soils with high available phosphorus, fast-growing copiotrophic taxa such as specific Proteobacteria rapidly assimilate these nutrients and outcompete the slower-growing Actinobacteria (Bongoua-Devisme et al., 2024). Nitrospirae, exhibited strong positive correlations with Zn, Cu, Cd, and Pb. This corroborates findings from highly contaminated mining areas, where Nitrospirae demonstrated remarkable tolerance to multiple heavy metals (Liu et al., 2022). This resilience is likely facilitated by specialized extracellular metal sequestration mechanisms such as polyphosphate complexation coupled with their unique chemolithoautotrophic metabolism, which allows them to outcompete less tolerant taxa under extreme stress (Lin et al., 2021). Similarly, we observed that Proteobacteria, Bacteroidetes, Verrucomicrobia, and Ascomycota were positively correlated with Zn and/or Cu (Deng et al., 2025; Tseng et al., 2021). The enrichment of these taxa suggests they harbor highly effective genetic determinants for heavy metal detoxification, such as P-type ATPases and multidrug efflux pumps, which confer a significant adaptive advantage by actively exporting toxic ions in the presence of severe localized pollution (Iimaa et al., 2025).

At the stain level, several most abundant Actinobacteria strains demonstrated acute sensitivity to heavy metal contamination. Specifically, *Conexibacter woesei* DSM 14684 and *Rubrobacter xylanophilus* DSM 9941 exhibited profound negative correlations with multiple heavy metals. *Conexibacter woesei* associated with protected and stable uncontaminated and forest soils (Galanova et al., 2025; Monciardini et al., 2003). For instance, (Salam et al., 2020) demonstrated that while *Conexibacter woesei* acts as keystone dominant species in healthy and uncontaminated soils, its abundance significantly reduced under high Cd overload. Our findings suggest, that suppression of *C. woesei* leaves a vacant ecological niche that is rapidly filled by other, more resilient taxa. Additionally, *Mesorhizobium amorphae* CCNWGS0123, *Rubrobacter xylanophilus*, and *Microvirga ossetica* similarly demonstrated strong negative correlations across the heavy metal spectrum. The parallel decline of these specific taxa under elevated metal concentrations suggests a shared vulnerability in their membrane permeability or an inability to upregulate heavy metal efflux pumps fast enough to outpace the cellular toxicity threshold (Iimaa et al., 2025; Nies, 2003). Conversely, strains enriched under heavy metal stress detected. *Bradyrhizobium* sp. SK17 and *Gemmatirosa kalamazoonesis* emerged as highly resilient taxa, exhibiting statistically significant positive correlations with Zn, Cu, Cd, and Pb. The enrichment of *Bradyrhizobium* species in heavily polluted zones corroborates recent findings by Agashe et al. (2024), who identified *Bradyrhizobium* as a predominant genus driving heavy metal resistance in long-term contaminated ecosystems. Furthermore, *Nocardioides dokdonensis* FR1436, *Pseudonocardia dioxanivorans* CB1190, *Modestobacter marinus* and *Rhodoplanes* sp. Z2-YC6860 displayed notable positive correlations with some of heavy mtals, indicating that heavy metal tolerance is distinctly strain-specific rather than universally conserved across phyla. These taxa actively replace sensitive relatives, driving a phenomenon of species replacement rather than a total loss of diversity under toxic stress (Tipayno et al., 2018). Notably absence of any significant correlations with alpha diversity indices, while beta diversity analyses show significant effect of HMs on microbial composition confirm this phenomenon. Our finding corroborates with other researches (Signorini et al., 2023; Shaw et al., 2020). Dimensional reduction analyses provide robust statistical evidence that heavy metal contamination exerts a profound, overarching effect on soil microbial communities. Emerged as the most significant independent driver shaping community structure, underscoring its acute localized toxicity and its role as a primary selective agent in these environments (Nikolova et al., 2025; Sun et al., 2025). Furthermore, the strong collinearity of Zn, Pb, Cd, Cu, H₂O, and P₂O₅ along the primary RDA axis suggests a shared anthropogenic origin deposition where these factors synergistically dictate the physicochemical landscape of the soil (Xiao et al., 2022). RDA biplots shows that, taxa such as *Conexibacter woesei* and *Microvirga ossetica* aligned in opposition to the heavy metal vectors, indicating their inability to survive extreme metal toxicity or outcompete more resilient microbes (Salam et al., 2020). Conversely, the strong positive association of *Bradyrhizobium icense* and *Modestobacter marinus* with the metal vectors confirms their ecological role as highly adapted “super-hosts.” These tolerant strains likely exploit specialized metabolic pathways and robust cellular defense mechanisms such as multidrug efflux pumps and active metal sequestration to aggressively colonize niches left vacant by the collapse of sensitive species (Agashe et al., 2024; Iimaa et al., 2025). The strong metal concentrations at Ridder and Ust-Kamenogorsk sites created extreme selective pressure that fundamentally restructured microbial assemblages while maintaining overall diversity indices comparable to less contaminated sites. Furthermore, the >80- year history of continuous industrial activity in East Kazakhstan has likely established stable metal-adapted communities where diversity is maintained through niche differentiation along micro-scale metal concentration gradients (Delgado-Baquerizo et al., 2016; Woszczyk et al., 2018).

The extensive diversity of both ARGs and MRGs identified in our study underscores the profound impact of chronic heavy metal pollution on the functional landscape of the soil resistome. The dominance of aminoglycoside, glycopeptide, and multidrug resistance classes highlights a robust, baseline defense repertoire within the soil microbial community. At the individual gene level, the high relative abundance of *vanR*, a key regulatory element for glycopeptide resistance, and *kdpE*, a two-component sensor kinase, underscores the critical role of highly sensitive environmental response systems. These regulatory genes allow soil bacteria to rapidly detect and adapt to external stressors, such as membrane-damaging agents and osmotic shifts induced by metal toxicity (Qi et al., 2021). Furthermore, the prominence of the *tetA*(48) tetracycline efflux pump highlights the reliance on active extrusion mechanisms for cellular defense (Wu et al., 2019). Importantly, the soil resistome was not evenly distributed but concentrated within specific bacterial hosts. *Conexibacter woesei* DSM 14684 and *Streptosporangium roseum* emerged as the primary ARG hosts. While *C. woesei* was previously shown to be sensitive to acute heavy metal toxicity, its role as a major ARG reservoir in baseline or moderately contaminated soils suggests it acts as a critical anchor for the native soil resistome before being outcompeted by metal-tolerant super-hosts under extreme stress. Spearman rank correlation analysis exposed complex co-occurrence patterns between resistance determinants and environmental drivers. Notably, the *ompR* and *bacA* genes exhibited statistically significant positive correlations with several heavy metals. The *ompR* gene acts as a global response regulator that directly controls the expression of outer membrane porins, altering cellular permeability, while *bacA* is involved in maintaining cell envelope integrity and undecaprenyl pyrophosphate synthesis (Ko and Choi, 2023; El Ghachi et al., 2004). Their positive correlation with heavy metals strongly implies a shared cross-resistance mechanism like genetic adaptations that reduce membrane permeability to limit the intracellular influx of toxic heavy metals concurrently prevent the entry of large antibiotic molecules, thereby conferring dual resistance to the host cell (Baker-Austin et al., 2006; Gillieatt and Coleman, 2024). In parallel with ARGs, the sampled soils harbored an extensive suite of 238 distinct MRGs, illustrating a deep-seated evolutionary response to chronic heavy metal exposure. The dominance of the *czcA* gene which encodes the central RND-type efflux pump for cadmium, zinc, and cobalt alongside *ruvB*, *arsM*, and *arsT*, points to a community heavily reliant on active metal extrusion and enzymatic detoxification pathways to maintain cellular homeostasis (Nies, 2003). Crucially, Spearman correlation analyses demonstrated that specific metal efflux and tolerance genes such as *silP*, *nczA*, *cusA/ybdE*, *ctpG*, *arsB*, and *copB*—were significantly and positively correlated with high concentrations of soil heavy metals. Genes such as *cusA* and *copB* are essential for copper extrusion, while *arsB* is a primary arsenite efflux pump (Silver and Phung, 1996). The strong statistical association between these specific MRGs and the physicochemical pollution gradient confirms that heavy metal contamination acts as a strict environmental filter. It exerts intense selective pressure, systematically enriching the microbiome for highly specific genetic determinants required to survive acute, localized inorganic toxicity (Thomas et al., 2020).

The alpha diversity analyses revealed deep interconnections between the taxonomic community and this functional resistance gene pool. The strong, highly significant positive correlations between taxonomic diversity estimators and both ARG and MRG diversity indices underscore that the soil resistome is fundamentally constrained by its microbial hosts. This robust coupling indicates that as the overall microbial community diversifies, it naturally sustains a broader, more diverse reservoir of functional resistance genes. This finding aligns with seminal literature and recent comprehensive metagenomic studies, which demonstrate that bacterial community composition and taxonomic richness act as the primary determinants shaping the architecture of soil resistomes (Forsberg et al., 2014; Rumi et al., 2025). Correlation analyses revealed that MRG alpha diversity is significantly and positively associated with all studied heavy metals as well as available phosphorus. This robust relationship indicates that elevated, persistent heavy metal loads exert intense selective pressure, directly driving the expansion and diversification of the metal resistance repertoire as the microbial community adapts to multi-metal toxicity (Xiao et al., 2025; Mi et al., 2025). Our findings demonstrate that the functional genetic repertoire actually expands to harbor a vast array of specialized survival strategies. Furthermore, the positive association with P_2_O_5_ suggests that macronutrient availability fuels the high metabolic costs. Specifically ATP required for efflux pumps and enzymatic detoxification is necessary for bacteria to maintain, express, and transfer these diverse resistance mechanisms (Tian et al., 2025). Interestingly, while ARG alpha diversity did not exhibit direct, significant correlations with heavy metal concentrations, it demonstrated highly significant positive correlations with all MRG diversity indices. This decoupling suggests that heavy metals do not directly induce ARG proliferation. Rather, the expansion of ARGs occurs as a secondary, collateral consequence of MRG selection. This strong ARG-MRG coupling provides compelling evidence for indirect co-selection. In these industrially impacted soils, heavy metal stress actively selects for microbial taxa that harbor mobile genetic elements such as plasmids and transposons, containing physically linked MRGs and ARGs, or organisms utilizing generalized defense mechanisms, such as multidrug efflux pumps capable of extruding both metals and antibiotics (Gillieatt and Coleman, 2024). Consequently, the heavy metal-driven adaptation of MRGs acts as the primary driver mechanism, facilitating the incidental co-occurrence, retention, and spread of clinical antibiotic resistance within agricultural and natural ecosystems (Zhao et al., 2019). The beta diversity analysis provides robust statistical evidence, that heavy metals significantly alter ARG and MRG diversity. We could suggest that heavy metal contamination acts as a strict environmental filter, fundamentally restructuring the functional resistome of the soil. RDA further isolated Zn and soil pH as the most dominant independent drivers of this variation. This aligns perfectly with recent metagenomic paradigms demonstrating that bioavailable heavy metals, particularly Zinc are not merely toxic agents but act as primary ecological drivers that dictate the beta diversity of soil ecosystems (Hou J. et al., 2025). Rather than causing a random loss of diversity, these metal gradients create highly specific, localized ecological niches that force a systematic, predictable shift in the genetic composition of both metal and antibiotic resistance profiles (Thomas et al., 2020). Perhaps the most critical finding from the RDA is the distinct directional alignment of multiple ARGs and MRGs with the heavy metal gradients. This overlapping trajectory, where MRGs and ARGs map precisely along the exact same metal vectors visually and statistically confirms that heavy metal pressure actively enriches for dual-resistance mechanisms. Because soil heavy metals are highly persistent and non-degradable, they exert continuous, multi-generational selective pressure. This persistent stress favors microbial hosts that carry MGEs containing physically linked ARGs and MRGs, or those expressing generalized cross-resistance mechanisms, such as multidrug efflux pumps capable of extruding both metals and antibiotics (Gillieatt and Coleman, 2024). Consequently, the spatial localization of heavy metal contamination not only shapes the immediate survival of the soil microbiome but acts as a potent, direct driver for the environmental dissemination and retention of clinical antibiotic resistance.

The primary microbial co-occurrence network exhibited a non-random, biological power-law distribution, characterized by significantly higher clustering and modularity compared to Erdős-Rényi null models. In microbial ecology, highly modular and clustered networks indicate intense niche partitioning and deterministic community assembly driven by severe environmental filtering (Zou et al., 2024). Under chronic heavy metal stress, the soil microbiome fragments into highly specialized functional modules that cooperate to survive localized inorganic toxicity. Within this network, topological analysis based on within-module (*Zi*) and among-module (*Pi*) connectivity identified specific keystone taxa essential for community cohesion. Notably, the majority of the identified Module Hubs belong to the phylum Actinobacteria. Actinobacteria are widely recognized for their profound genomic plasticity, extensive secondary metabolite production, and intrinsic resistance capabilities. Their emergence as primary topological anchors confirms their vital ecological role in maintaining overall network stability, structural cohesion, and functional redundancy in highly toxic, heavily impacted environments (Li et al., 2023). To directly investigate the co-selection of resistance, the tripartite association network which maps strains, MRGs, and ARGs revealed that the mobilization of the resistome is heavily reliant on a few critical biological reservoirs. *Bradyrhizobium icense* and *Conexibacter woesei* exhibited the highest degree centralities, functioning as primary “super-hosts.” These taxa actively bridge diverse resistance determinants, allowing them to rapidly adapt and dominate heavily contaminated niches. This network was structurally dominated by highly disseminated core resistance genes. The extreme centrality of specific MRGs like *acn*, *ruvB*, *furA* alongside widely distributed ARGs like *vanR*, *rpoB2*, *tetA(48)* within shared microbial hosts provides profound visual and statistical evidence for co-selection. The fact that these specific genes cross-connect through the same keystone species suggests that they are likely physically linked on the same mobile genetic elements such as plasmids, integrons, or co-regulated by the same global stress-response networks (Liu et al., 2024; Mazhar et al., 2021). The targeted bipartite ARG-MRG network further isolated and confirmed the intense co-selection dynamics occurring in these soils. Like the broader community network, this sub-network maintained a highly connected, scale-free topology with elevated modularity, indicating that the genetic linkage between metal and antibiotic resistance is highly structured rather than random. The extreme centrality of specific MRGs like *acn*, *ruvB*, *furA* alongside widely distributed ARGs like *vanR*, *rpoB2*, *tetA48* within shared microbial hosts provides profound visual and statistical evidence for co-selection. The fact that these specific genes cross-connect through the same keystone species suggests that they are likely physically linked on the same mobile genetic elements such as plasmids, integrons, or co-regulated by the same global stress-response networks (Partridge et al., 2018). Metagenomic surveys of metal-impacted systems further show that integrons, transposases and plasmid-associated transfer genes increase in abundance along heavy metal gradients, and that integron-associated ARGs frequently co-vary with *czcA*-like and other metal efflux determinants, consistent with MGE-mediated co-selection (Pal et al., 2015; Bhat et al., 2023). The peak connectivity of specific MRG hubs such as *copS* and *corA*, which regulate heavy metal sensing and transport with ARG hubs such as *vanH* and the *tetA(48)* efflux pump illustrates a synergistic survival strategy. The abundance of significant correlations between these gene classes highlights that heavy metal contamination acts as the primary evolutionary driver. By selecting for metal extrusion and tolerance mechanisms, the heavy metal gradient inadvertently but systematically accelerates the co-transfer, environmental retention, and proliferation of clinical antibiotic resistance within the soil microbiome (Vats et al., 2022; Heydari et al., 2023).

Functional profiling indicates that heavy metal contamination restructures, rather than collapses, the metabolic potential of soil microbiomes, with communities retaining core energy production and nutrient-cycling functions while down-regulating broad transport and transcriptional capacity under high metal loads. The dominance of transport systems, energy production and conversion, and amino−/carbohydrate-metabolism in both KEGG and COG annotations is consistent with previous metagenomic studies of contaminated soils, where stress-tolerant communities maintain minimal metabolic repertoires needed for survival in toxic environments (Hur and Park, 2019; Feng et al., 2018; Hemme et al., 2010). The strong negative correlations of multiple ABC-2 type transporters, branched-chain amino acid and peptide/nickel transport systems, and RNA polymerase subunits/sigma factors with Zn, Pb, and Cd suggest that increasing metal loads suppress generalized nutrient uptake and transcription in favor of more specialized metal homeostasis and detoxification pathways (Fajardo et al., 2019). Similar suppression of broad transport and information-processing functions, coupled with enrichment of metal-resistance and ion-transport genes, has been reported in Cd- and mixed-metal-contaminated soils, supporting the view that chronic metal exposure selects for metabolically conservative, stress-adapted taxa (Feng et al., 2018; Li et al., 2023). The observed negative association of cytoskeleton-related COGs with Cu and Cd, together with positive correlations between inorganic ion transport and Zn, further reinforce that cell structure and ion-handling systems are key functional targets of metal-driven selection (Dutta et al., 2018; Bongoua-Devisme et al., 2024). Overall, the concordant KEGG and COG patterns indicate that Zn, Pb, and Cd gradients impose strong functional filters that favor communities with robust defense mechanisms, inorganic ion transport, and regulatory plasticity, while reducing investment in broad transport and transcriptional machinery. These findings align with genome-resolved work showing that long-term metal contamination enriches keystone taxa carrying dense complements of metal-resistance genes and plant-beneficial traits (Li et al., 2023). This finding highlighs their potential for microbe-assisted remediation and plant-microbiome-based strategies in contaminated soils (Jia et al., 2025).

This study has several limitations that should be considered when interpreting the results. First, although Oxford Nanopore long-read sequencing enabled strain-level and genome-resolved insights, the platform’s higher per-base error rate relative to short-read technologies may have affected the sensitivity and specificity of ARG and MRG detection, as well as functional annotation, despite the use of stringent quality-control and alignment thresholds. Second, our inferences about resistance and metabolism are based on gene abundances derived from DNA-level metagenomes. We did not measure gene expression or enzyme activities, so we cannot distinguish between actively expressed resistance mechanisms and dormant or lowly expressed genes, nor can we directly link functional shifts to physiological responses under metal stress. Third, the study design was cross-sectional, capturing a single time point in systems with more than 80 years of industrial activity, and thus cannot resolve temporal dynamics, seasonal variability, or the pace of community adaptation to changing metal loads. Fourth, heavy metals, nutrients, and other soil physicochemical properties were strongly collinear in this industrial context, and we did not quantify co-occurring organic pollutants or antibiotics, so some of the associations we attribute to metal selection may be partially confounded by unmeasured co-stressors and land-use history. The inability to account for seasonal variability was due to logistical and operational constraints, including limited for repeated field sampling and high-throughput sequencing across multiple time points. Fifth, our analyses relied on existing reference databases, which may underrepresent novel taxa and resistance determinants in chronically polluted soils, and we did not explicitly reconstruct or validate mobile genetic elements that physically link ARGs and MRGs. That limits our ability to prove genetic co-localization rather than statistical co-occurrence. Finally, the assembly-free analytical workflow, while maximizing read utilization and avoiding assembly bias, does not yield assembled contigs, bins, or plasmid sequences. As a result, study cannot confirm physical co-localization of ARGs and MRGs on the same genetic element at the replicon level. Future works employing assembly, metagenomic binning, and plasmid identification on these long-read datasets would allow direct verification of whether the co-occurring ARGs and MRGs identified here are physically co-located on the same plasmid or transposon; Hi-C linkage, metatranscriptomics, and targeted cultivation experiments along controlled metal and antibiotic gradients will be essential to validate the inferred co-selection mechanisms and to better quantify their implications for environmental and clinical antimicrobial resistance.

## 5 Conclusion

Using genome-resolved long-read metagenomics, this study shows that chronic heavy metal contamination in industrial soils of East Kazakhstan maintains high overall microbial and resistome diversity while fundamentally reshaping community composition at the strain and functional levels. Heavy metals, with zinc emerging as the dominant driver and soil pH as a key modifier, exert strong environmental filtering that promotes species replacement rather than richness loss and reorganizes both ARG and MRG assemblages along localized pollution gradients. Our analyses reveal that persistent metal stress expands and diversifies the metal resistance repertoire and that ARG diversity tracks this expansion through tight coupling to MRG diversity, rather than directly to metal concentrations, supporting an indirect co-selection mechanism. Genomic association and co-occurrence networks further demonstrate that the resistome is highly non-random, scale-free, and modular, with a limited set of metal-tolerant Actinobacteria and *Bradyrhizobium* strains acting as “super-hosts” that physically and ecologically connect widely disseminated MRG hubs with key glycopeptide, tetracycline, and multidrug resistance determinants. These findings position long-term metal-polluted industrial soils as important environmental reservoirs and amplifiers of clinically relevant antibiotic resistance, even in the absence of documented direct antibiotic inputs. They underscore the need to explicitly incorporate heavy metals and co-selected MRG–ARG modules into One Health antimicrobial resistance risk frameworks and monitoring programs. In parallel, the identified metal-adapted keystone taxa and resistance hubs represent promising targets and biomarkers for microbe-assisted remediation and for tracking the effectiveness of interventions in contaminated landscapes. Future work combining plasmid- and MGE-resolved long-read assembly, Hi-C linkage, metatranscriptomics, and controlled gradient experiments will be essential to validate physical co-localization, quantify *in situ* expression of co-selected resistance genes, and better predict the transfer risk of environmental ARGs into clinical and agricultural settings.

## Data Availability

The raw Oxford Nanopore metagenomic sequencing reads generated in this study are available in the NCBI Sequence Read Archive (SRA) under BioProject accession number PRJNA1436833.

## Footnotes

1 https://www.dropbox.com/scl/fi/ntldm1ua1etwwlzwyqyl5/refseq_with_annotations.tar.gz?rlkey=6dhgg4132ypllo0k32zjx4m1g&e=1&dl=0, accessed on 13 February 2026.
