# Supplementary material for: Genome-resolved metagenomics reveals co-selection of antibiotic and metal resistance in chronically polluted industrial soils
Source: Front Microbiol. 2026 May 8;17:1829529. doi: 10.3389/fmicb.2026.1829529 (PMC13194108; doi:10.3389/fmicb.2026.1829529)
Supplement: Supplementary file 1 [file Table_1.DOCX]

Supplementary Material

## Supplementary Figures


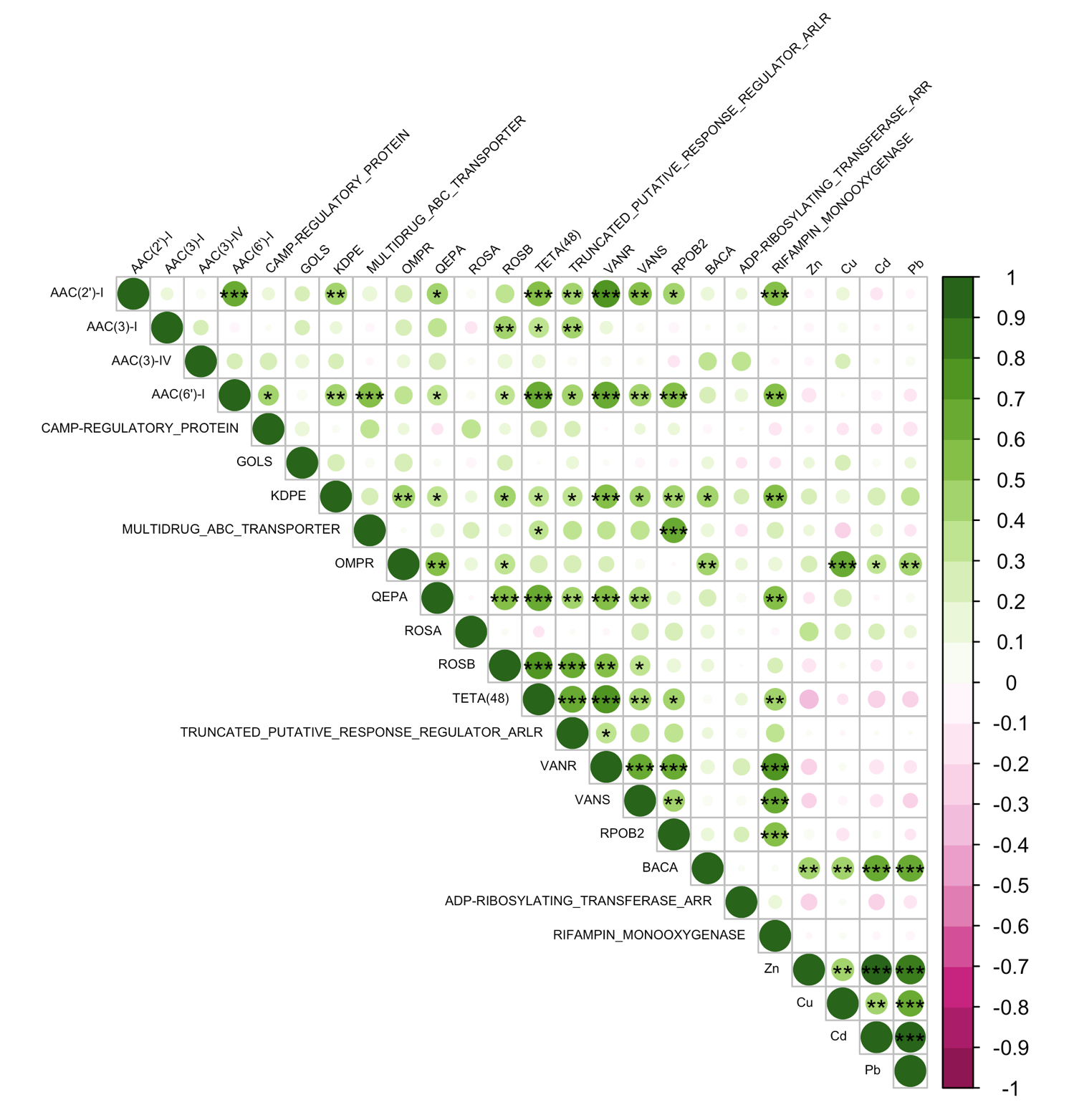


**Supplementary Figure 1.** Correlation plot of top ARGs, HMs and soil physiochemical properties. Statistical significance is denoted by asterisks: * p < 0.05, ** p < 0.01, *** p < 0.001.


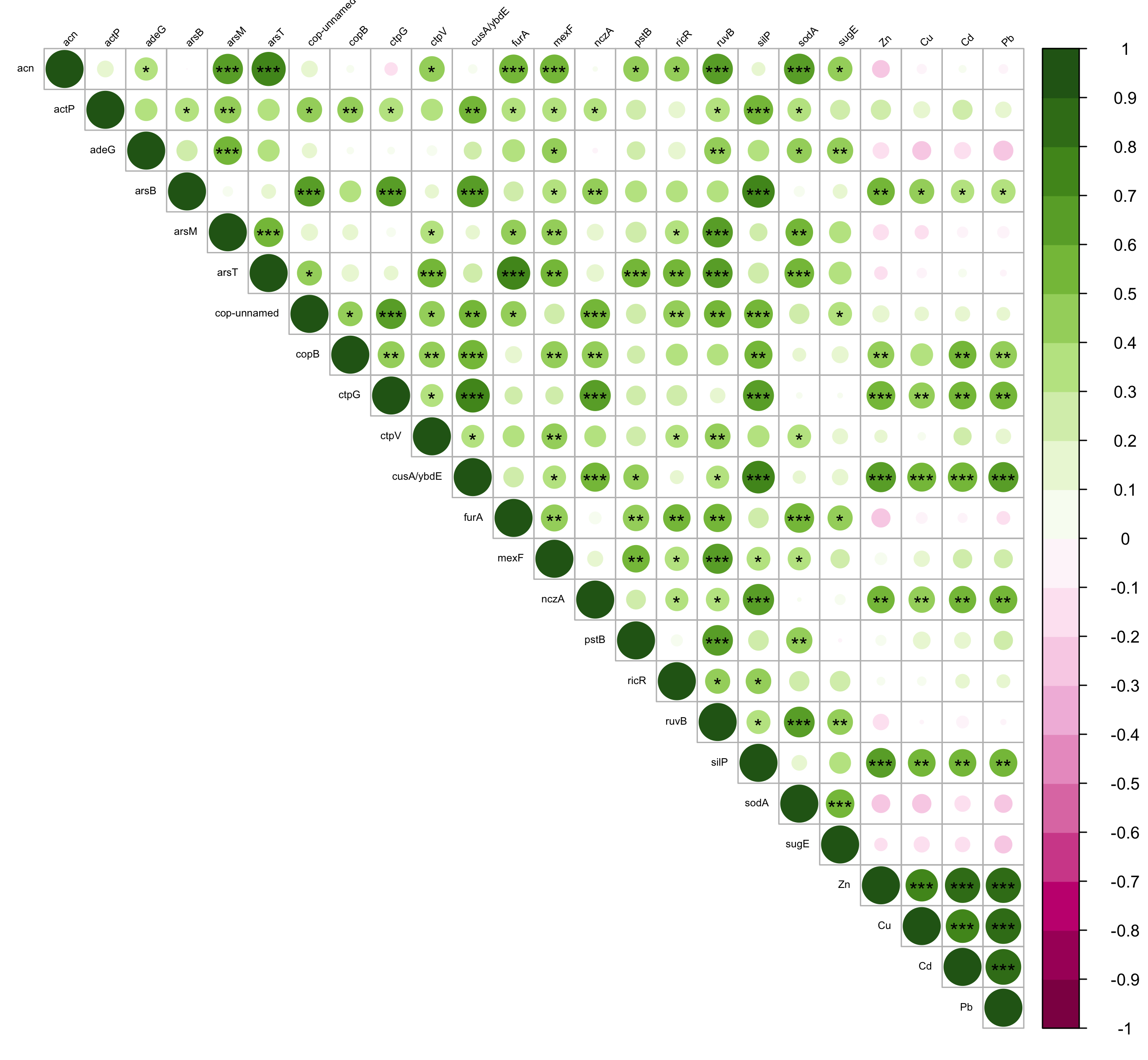


**Supplementary Figure 2.** Correlation plot of top MRGs, HMs and soil physiochemical properties. Statistical significance is denoted by asterisks: * p < 0.05, ** p < 0.01, *** p < 0.001.


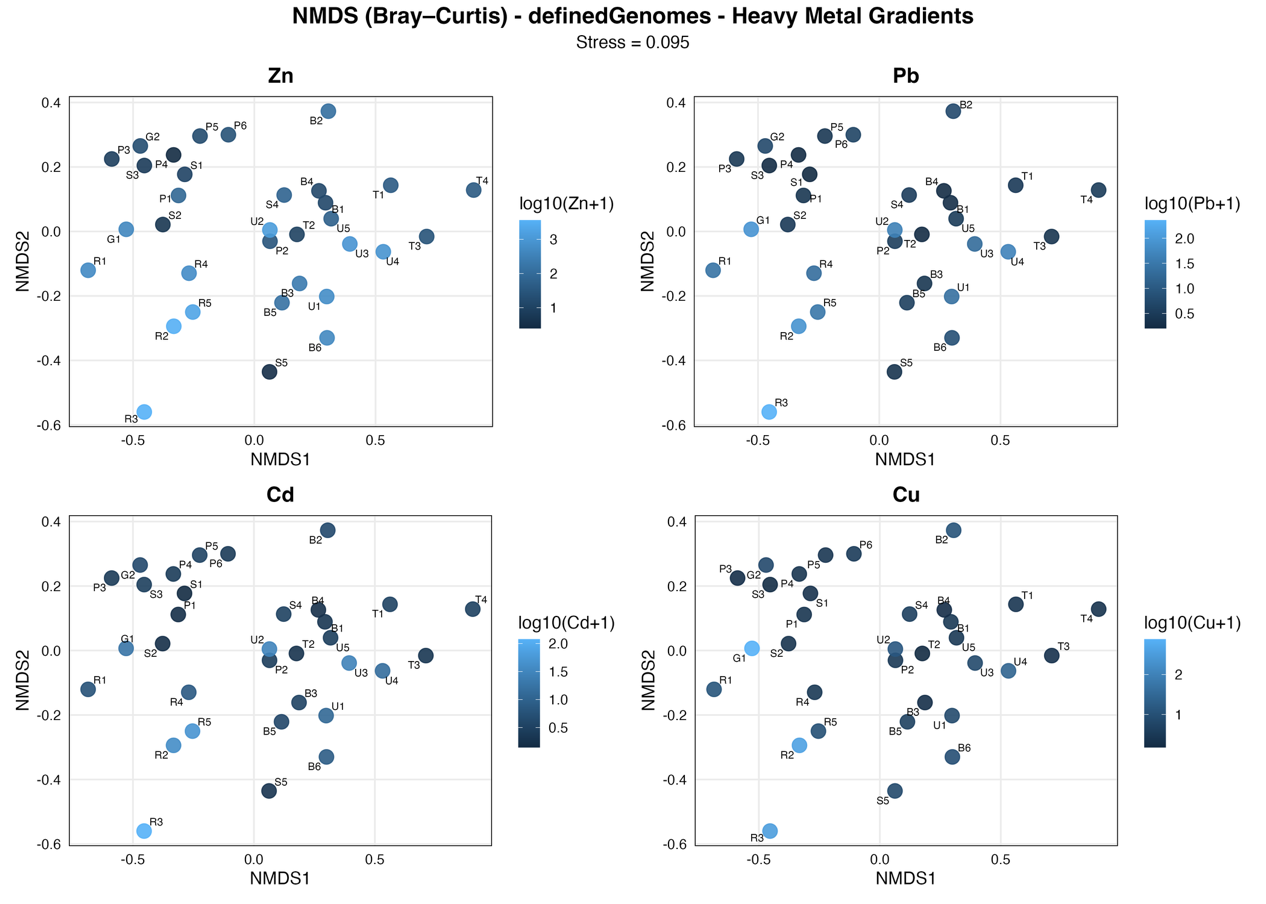

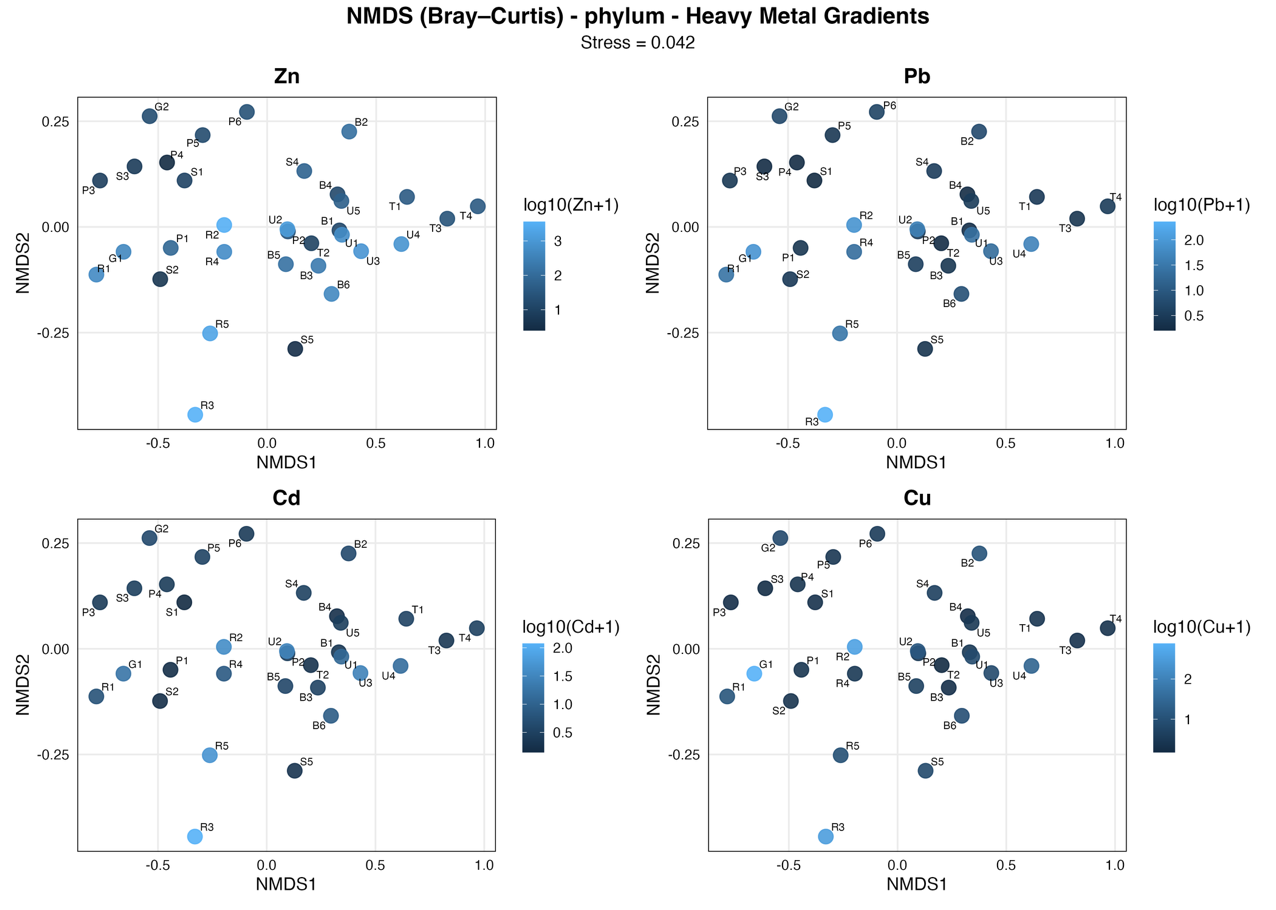


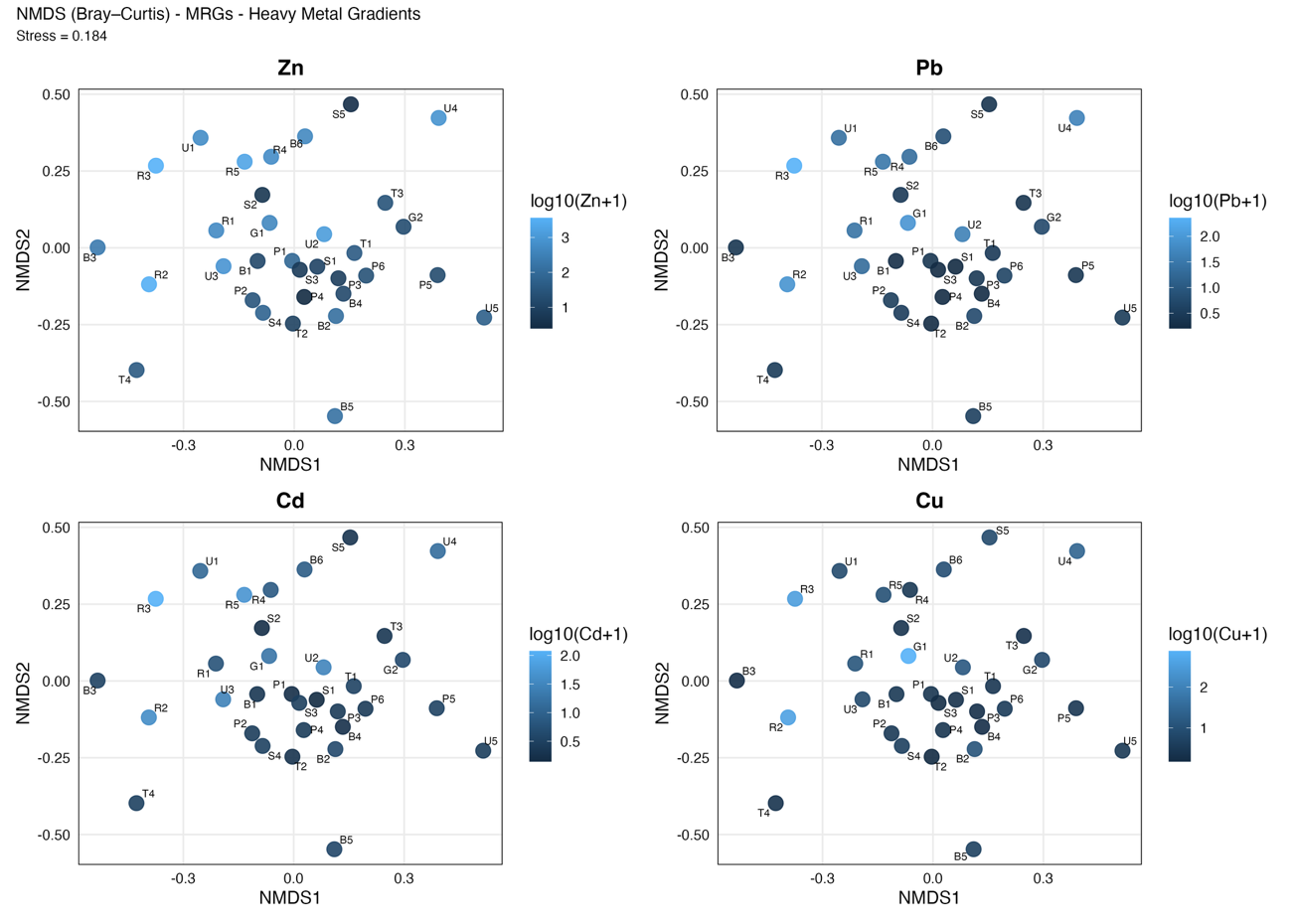
**
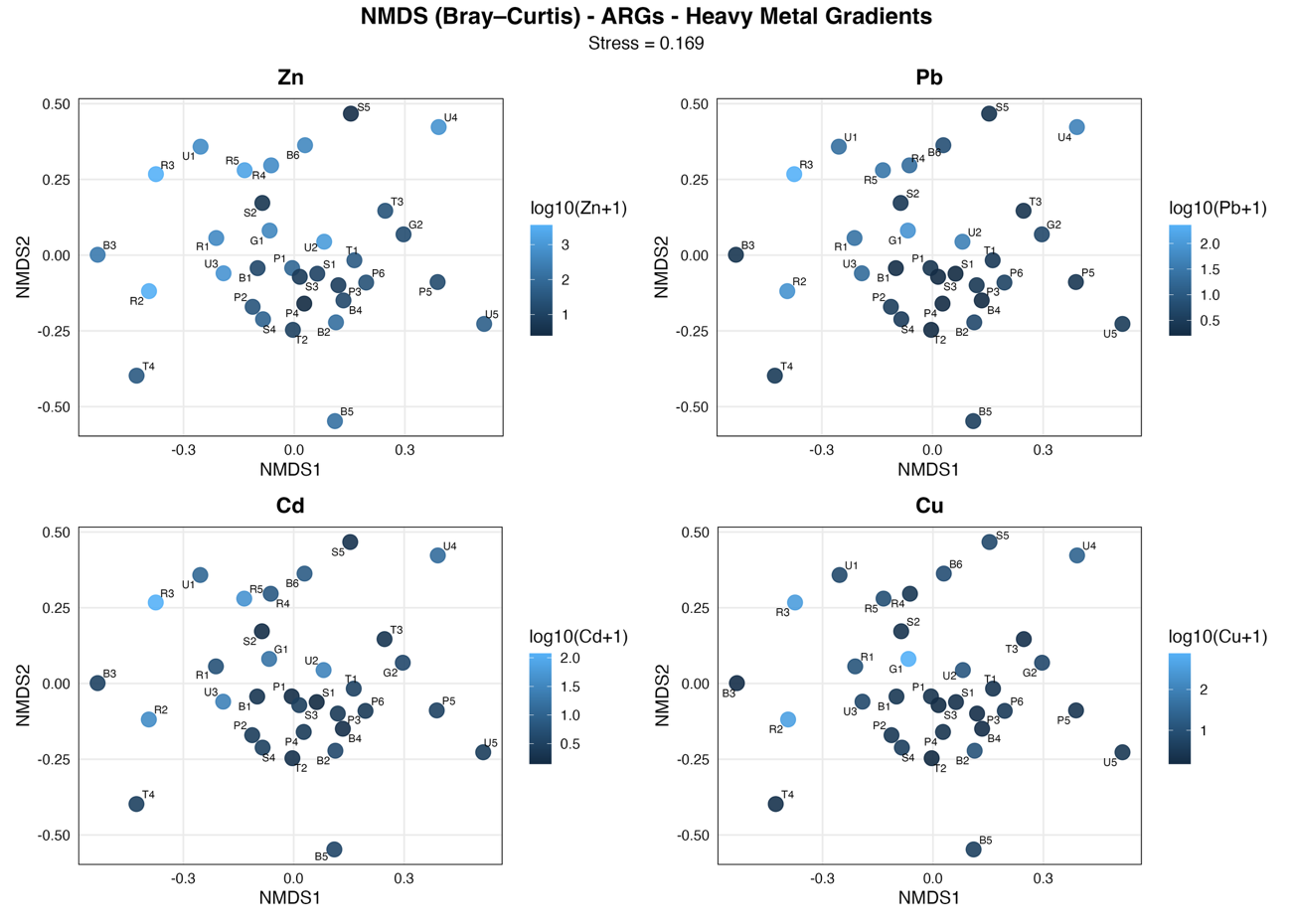
**

**Supplementary Figure 3.** NMDS ordination plot with the log-transformed HM values. (a) NMDS at phylum level;(b) NMDS at strain level; (c) NMDS for ARG communities; (d) NMDS for MRG communities.


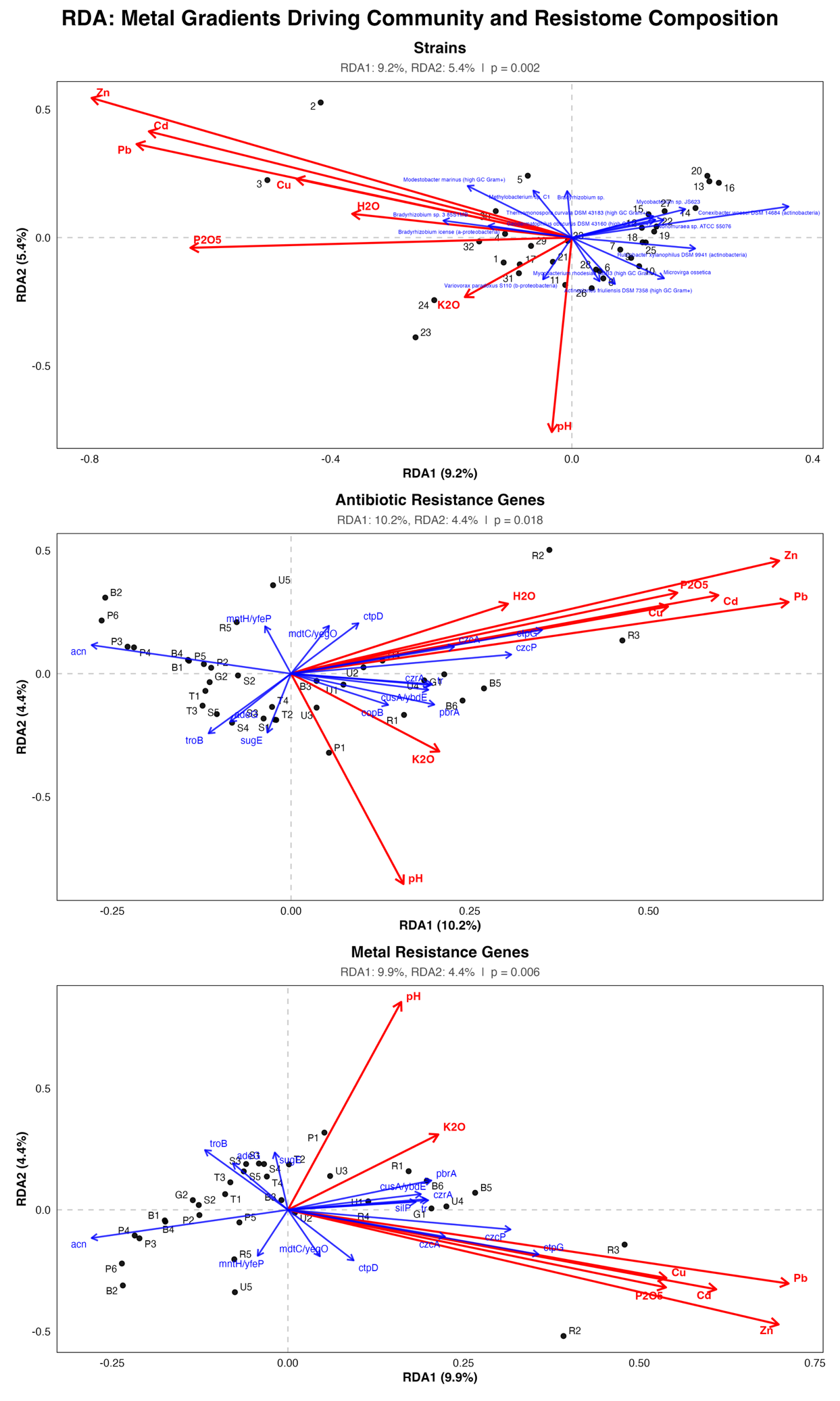


**Supplementary Figure 4.** RDS analysis of communities with Vectors of soil Physiochemical properties and heavy Metals. Global anova test with heavy metal results; (a) RDA for strain level communities; (b) RDA for ARGs matrix; (C) RDA for MRGs matrix.

##
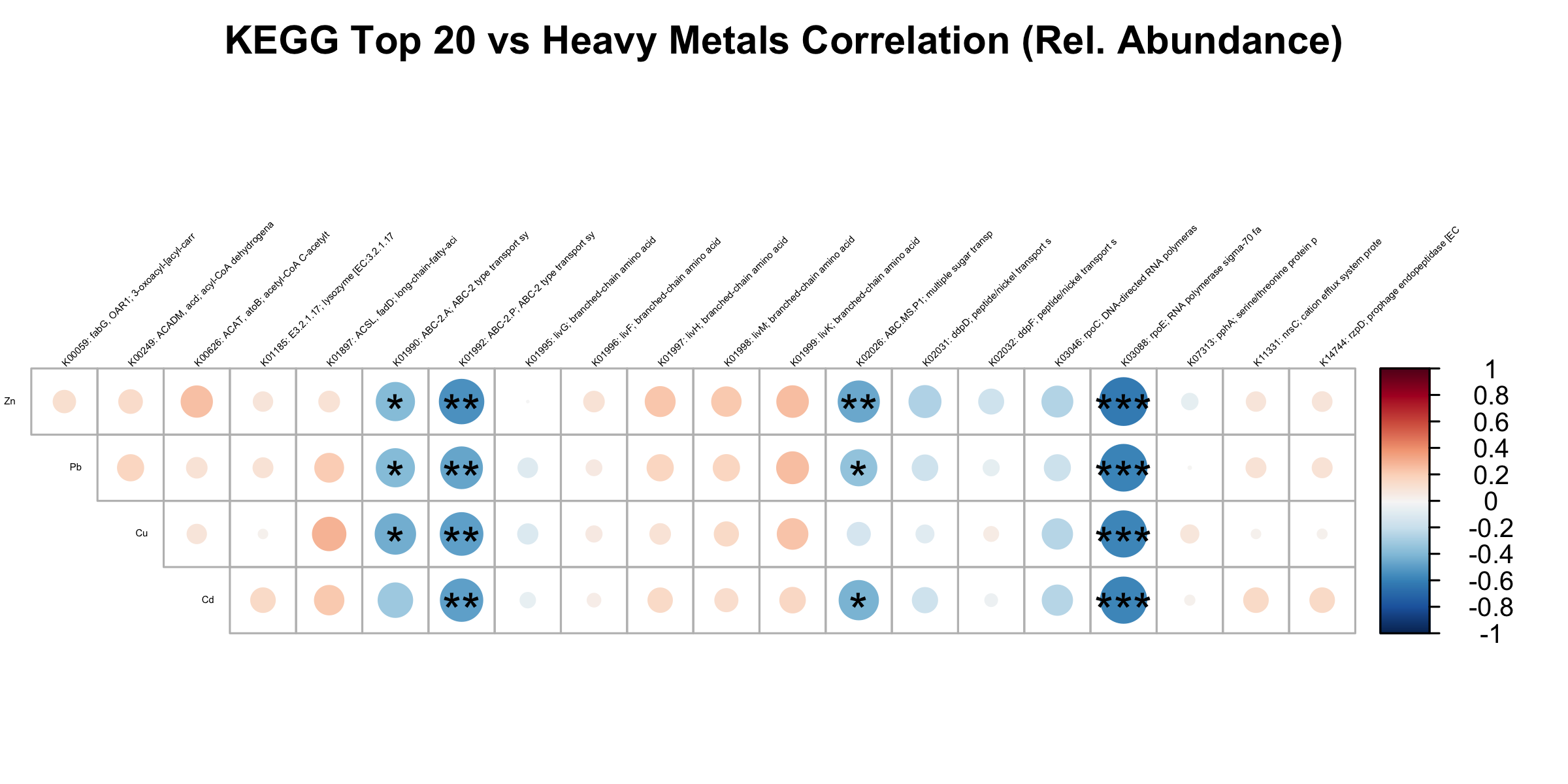

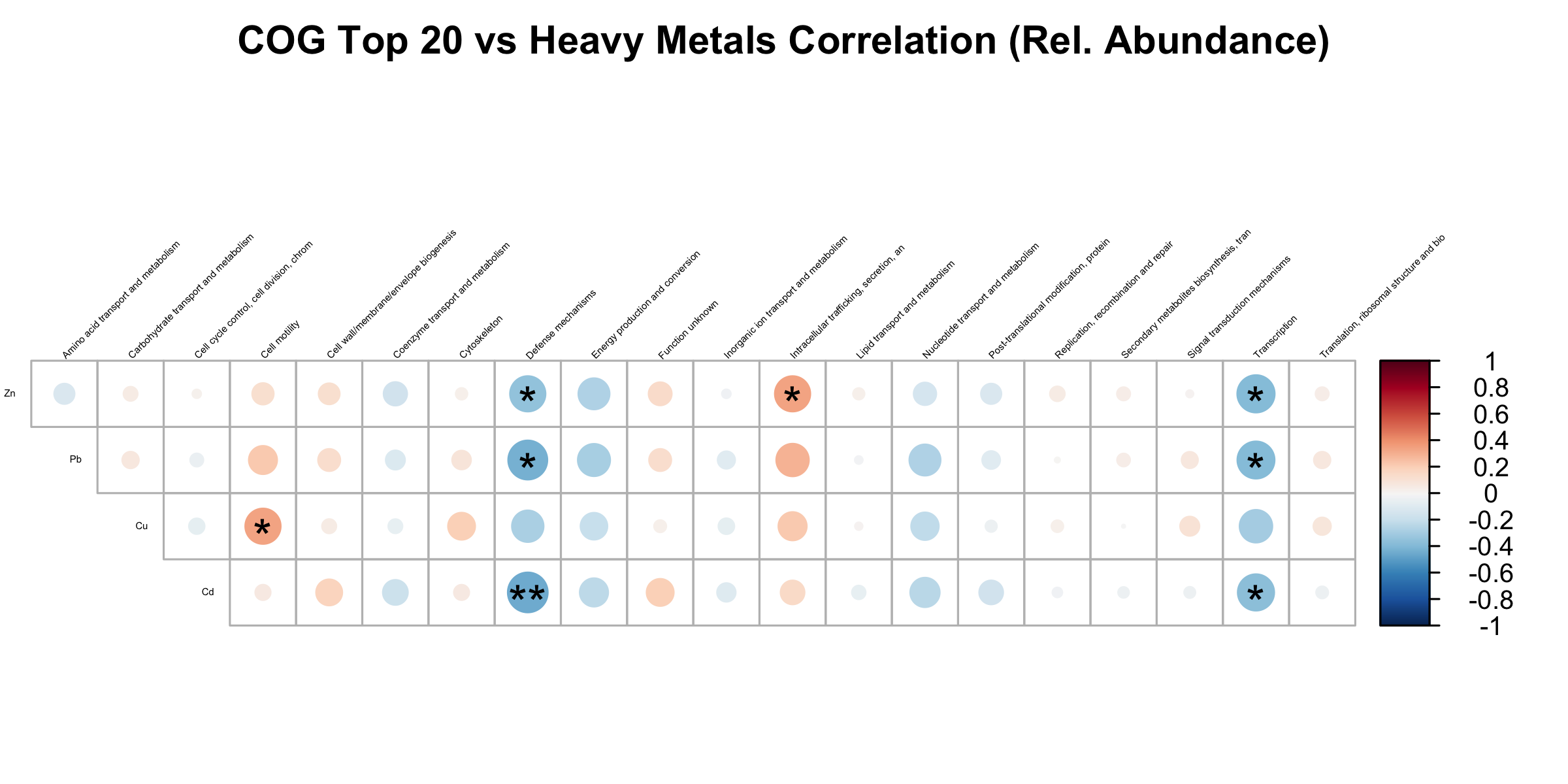


**Supplementary Figure 5.** Spearman correlation plot of Top functional annotation pathways with HMs.(a) Correlation with KEGG annotations; (b) Correlation with COG annotations

##
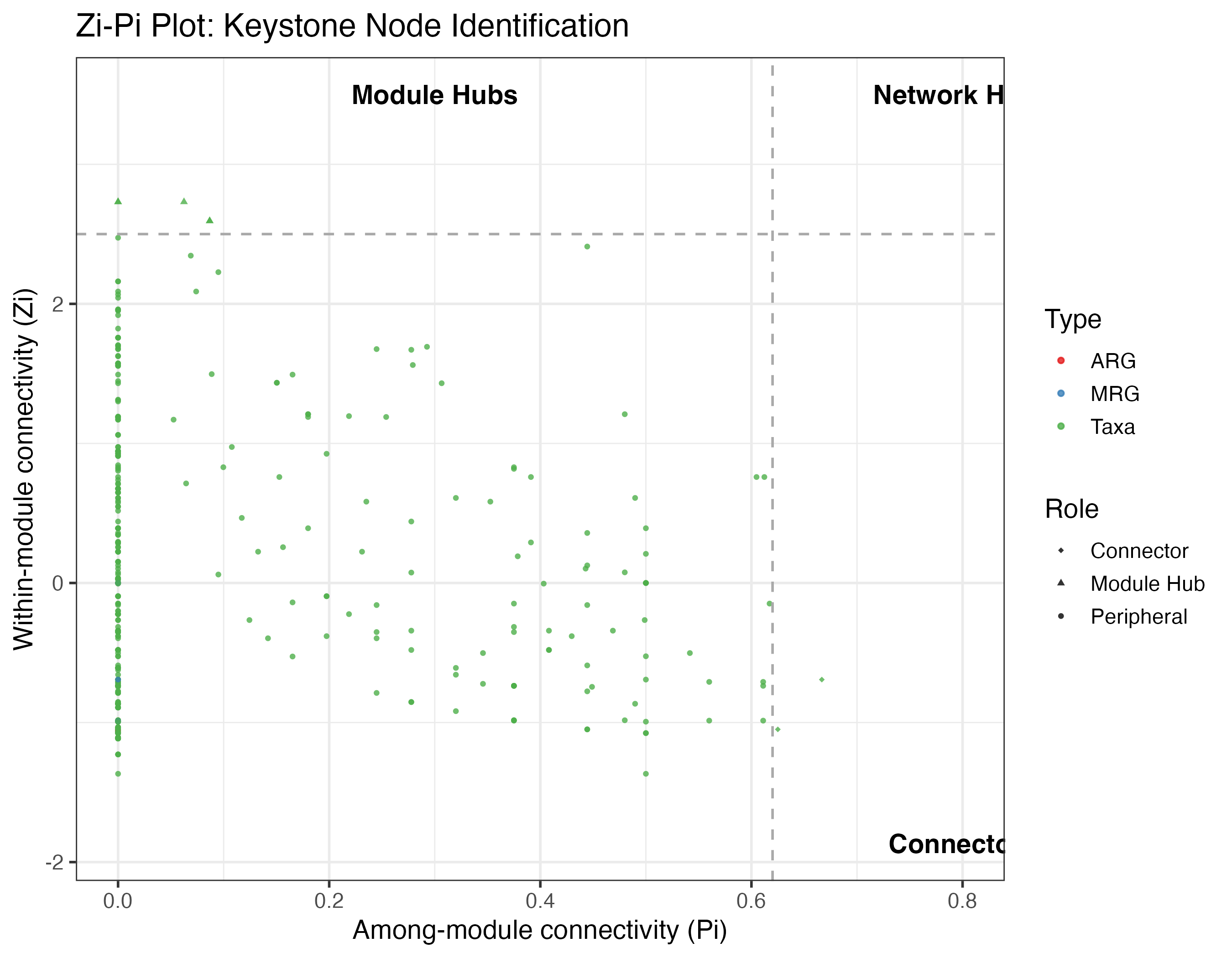


**Supplementary Figure 6.** Zi–Pi plot illustrating within‑module connectivity (Zi) versus among‑module connectivity (Pi) for nodes in the ARG–MRG–taxa co‑occurrence network, used to identify keystone nodes.

## Supplementary tables

| № | Localization | N2 | P2O5 | K2O | pH | Zn | Cu | Cd | Pb | H2O |  | Coordinates  (Longitude/Latitude) | |
| --- | --- | --- | --- | --- | --- | --- | --- | --- | --- | --- | --- | --- | --- |
| R1 | Ridder | 81.2 | 32 | 500 | 7.2 | 518 | 11.3 | 4.8 | 25.2 | 1.64 |  | 50.35228 | 83.51445 |
| R2 | Ridder | 67.2 | 112 | 210 | 5.77 | 3276 | 392 | 39 | 82 | 2.24 |  | 50.35231 | 83.50495 |
| R3 | Ridder | 47.6 | 214 | 420 | 5.93 | 3624 | 328 | 118 | 227 | 3 |  | 50.35086 | 83.48562 |
| R4 | Ridder | 70 | 98 | 680 | 6.21 | 578 | 1.9 | 6.4 | 20.2 | 3.28 |  | 50.34166 | 83.49445 |
| R5 | Ridder | 165.2 | 202 | 630 | 5.57 | 1935 | 8.6 | 48 | 29.4 | 2.96 |  | 50.36110 | 83.50329 |
| S1 | Shemonaikha | 50.4 | 16 | 480 | 7.09 | 9.9 | 1.2 | 0.4 | 0.6 | 2.26 |  | 50.59161 | 81.88157 |
| S2 | Shemonaikha | 75.6 | 18 | 280 | 6.66 | 3.8 | 1.4 | 0.6 | 1.9 | 3.1 |  | 50.58868 | 81.91599 |
| S3 | Shemonaikha | 56 | 14 | 330 | 7.24 | 5.7 | 0.7 | 1.9 | 0.7 | 2.18 |  | 50.58402 | 81.87749 |
| S4 | Shemonaikha | 42 | 24 | 470 | 7.1 | 64 | 3.2 | 2.2 | 2.3 | 0.58 |  | 50.54346 | 81.85400 |
| S5 | Shemonaikha | 36.4 | 6 | 160 | 7.35 | 2.1 | 6 | 1 | 1.3 | 0.48 |  | 50.55759 | 81.84727 |
| P1 | Pervomaisk | 47.6 | 24 | 750 | 7.3 | 81 | 1.5 | 0.6 | 1.5 | 1.96 |  | 50.27801 | 82.00498 |
| P2 | Pervomaisk | 89.6 | 52 | 530 | 6.21 | 27.8 | 2 | 1.7 | 2.5 | 2.42 |  | 50.29331 | 82.01324 |
| P3 | Pervomaisk | 61.6 | 46 | 710 | 5.56 | 7.1 | 0.9 | 1.5 | 1.7 | 1.92 |  | 50.27764 | 82.01912 |
| P4 | Pervomaisk | 50.4 | 22 | 240 | 6.18 | 1.5 | 1.5 | 1.6 | 0.7 | 1.84 |  | 50.27325 | 82.02766 |
| P5 | Pervomaisk | 187.6 | 136 | 950 | 5.66 | 15.9 | 1.7 | 2.4 | 1.6 | 1.12 |  | 50.26831 | 82.01295 |
| P6 | Pervomaisk | 58.8 | 42 | 240 | 5.77 | 23.6 | 2.5 | 1.5 | 3.4 | 1.28 |  | 50.26299 | 82.07384 |
| G1 | Glubokoe | 30.8 | 76 | 450 | 6.61 | 442 | 762 | 17 | 85 | 1.94 |  | 50.13480 | 82.31807 |
| G2 | Glubokoe | 47.6 | 22 | 350 | 6.61 | 17.5 | 5.8 | 3.1 | 4.2 | 2.38 |  | 50.16155 | 82.30049 |
| B1 | Belousovka | 47.6 | 16 | 210 | 6.48 | 12.4 | 1.9 | 1.1 | 0.9 | 2.6 |  | 50.13000 | 82.47115 |
| B2 | Belousovka | 70 | 76 | 210 | 5.52 | 120 | 10.5 | 3.1 | 4.3 | 1.44 |  | 50.13005 | 82.48750 |
| B3 | Belousovka | 64.4 | 82 | 530 | 6.6 | 224 | 0.9 | 2.4 | 1.5 | 3.14 |  | 50.12204 | 82.49545 |
| B4 | Belousovka | 50.4 | 18 | 280 | 6.38 | 13 | 0.8 | 0.7 | 0.7 | 2.62 |  | 50.11832 | 82.50514 |
| B5 | Belousovka | 78.4 | 288 | 810 | 6.69 | 139 | 3.8 | 2.8 | 2.7 | 2.14 |  | 50.12392 | 82.51152 |
| B6 | Belousovka | 106.4 | 202 | 990 | 6.56 | 453 | 8.1 | 7.1 | 6 | 3.42 |  | 50.13008 | 82.51066 |
| T1 | TMK | 50.4 | 44 | 420 | 6.68 | 39.2 | 1.6 | 2.4 | 1.9 | 1.1 |  | 50.02666 | 82.74874 |
| T2 | TMK | 42 | 42 | 250 | 7.42 | 9 | 0.5 | 0.9 | 0.7 | 0.98 |  | 50.02682 | 82.77135 |
| T3 | TMK | 170.8 | 48 | 1080 | 6.03 | 26.1 | 1.1 | 1.1 | 1.4 | 1.42 |  | 50.03651 | 82.77000 |
| T4 | TMK | 72.8 | 54 | 480 | 6.92 | 37.8 | 1.3 | 2.2 | 2.2 | 1.92 |  | 50.03692 | 82.76096 |
| U1 | Ust-Kamenogorsk | 72.8 | 108 | 560 | 6.64 | 591 | 6.4 | 10.9 | 22.9 | 1.14 |  | 50.03403 | 82.73848 |
| U2 | Ust-Kamenogorsk | 36.4 | 50 | 620 | 6.23 | 1255 | 12.9 | 28.8 | 42 | 4.9 |  | 50.00077 | 82.58354 |
| U3 | Ust-Kamenogorsk | 112 | 168 | 640 | 6.81 | 862 | 5.9 | 27 | 19 | 1.62 |  | 49.98743 | 82.63815 |
| U4 | Ust-Kamenogorsk | 67.2 | 82 | 440 | 6.8 | 818 | 24.1 | 13.1 | 45 | 1.18 |  | 49.97572 | 82.60204 |
| U5 | Ust-Kamenogorsk | 100.8 | 210 | 330 | 5.49 | 52 | 1.9 | 1.8 | 2.2 | 3.34 |  | 49.98978 | 82.58381 |

**Supplementary Table 1.** Level of Soil Physiochemical properties and HMs across all samples and sampling sitesof soils.

| Variable | Mean | SD | SE | CV | Min | Q1 | Median | Q3 | Max | Range | IQR | Skewness | Kurtosis |
| --- | --- | --- | --- | --- | --- | --- | --- | --- | --- | --- | --- | --- | --- |
| N2 | 72.97 | 38.23 | 6.66 | 52.39 | 30.80 | 47.60 | 64.40 | 78.40 | 187.60 | 156.80 | 30.80 | 1.70 | 2.31 |
| P2O5 | 80.12 | 73.03 | 12.71 | 91.15 | 6.00 | 24.00 | 50.00 | 108.00 | 288.00 | 282.00 | 84.00 | 1.24 | 0.58 |
| K2O | 491.82 | 239.24 | 41.65 | 48.64 | 160.00 | 280.00 | 470.00 | 630.00 | 1080.00 | 920.00 | 350.00 | 0.74 | -0.12 |
| pH | 6.46 | 0.58 | 0.10 | 9.00 | 5.49 | 6.03 | 6.60 | 6.81 | 7.42 | 1.93 | 0.78 | -0.15 | -1.03 |
| Zn | 463.16 | 883.41 | 153.78 | 190.73 | 1.50 | 13.00 | 52.00 | 518.00 | 3624.00 | 3622.50 | 505.00 | 2.57 | 5.90 |
| Cu | 48.91 | 154.55 | 26.90 | 316.00 | 0.50 | 1.40 | 2.00 | 8.10 | 762.00 | 761.50 | 6.70 | 3.63 | 12.74 |
| Cd | 10.82 | 22.51 | 3.92 | 208.01 | 0.40 | 1.50 | 2.40 | 7.10 | 118.00 | 117.60 | 5.60 | 3.61 | 13.93 |
| Pb | 19.53 | 43.16 | 7.51 | 220.95 | 0.60 | 1.50 | 2.30 | 20.20 | 227.00 | 226.40 | 18.70 | 3.69 | 14.55 |
| H2O | 2.1 | 0.95 | 0.17 | 45.18 | 0.48 | 1.42 | 1.96 | 2.62 | 4.90 | 4.42 | 1.20 | 0.62 | 0.53 |

**Supplementary Table 2.** Descriptive statistics of Soi Physiochemical properties and HMs across all samples

| Sample | Tax Shannon | Tax Simpson | Tax_Chao1 | Tax ACE | Tax Richness | Tax Evenness | Tax Total Abunance | ARG Shannon | ARG Simpson | ARG Chao1 | ARG ACE | ARG Richness | ARG Evenness | ARG Total ARGs | MRG Shannon | MRG Simpson | MRGChao1 | MRG ACE | MRG Richness | MRG Evenness | MRG Tota lMRGs |
| --- | --- | --- | --- | --- | --- | --- | --- | --- | --- | --- | --- | --- | --- | --- | --- | --- | --- | --- | --- | --- | --- |
| B1 | 5.24 | 0.98 | 1687.11 | 1772.653 | 1290 | 0.73 | 20790 | 2.91 | 0.93 | 46.75 | 47.22 | 24 | 0.92 | 50 | 3.77 | 0.96 | 96.31 | 96.75 | 76 | 0.87 | 293 |
| B2 | 4.85 | 0.98 | 1199.91 | 1297.93 | 952 | 0.71 | 19429 | 2.89 | 0.93 | 36.00 | 43.93 | 23 | 0.92 | 45 | 3.43 | 0.92 | 89.06 | 109.77 | 60 | 0.84 | 160 |
| B3 | 5.22 | 0.98 | 1780.72 | 1885.08 | 1356 | 0.72 | 23328 | 3.10 | 0.95 | 43.43 | 51.20 | 24 | 0.98 | 32 | 4.00 | 0.97 | 127.59 | 134.61 | 84 | 0.90 | 258 |
| B4 | 5.14 | 0.98 | 1584.14 | 1717.66 | 1221 | 0.72 | 21158 | 2.50 | 0.89 | 30.75 | 43.51 | 17 | 0.88 | 35 | 3.61 | 0.95 | 79.00 | 87.39 | 64 | 0.87 | 222 |
| B5 | 5.30 | 0.98 | 1788.44 | 1870.20 | 1399 | 0.73 | 27378 | 2.95 | 0.94 | 53.00 | 55.22 | 23 | 0.94 | 37 | 3.90 | 0.97 | 152.46 | 153.72 | 83 | 0.88 | 272 |
| B6 | 5.27 | 0.98 | 1748.15 | 1870.34 | 1319 | 0.73 | 21868 | 2.81 | 0.93 | 32.20 | 34.96 | 19 | 0.95 | 30 | 4.02 | 0.97 | 127.18 | 131.14 | 88 | 0.90 | 300 |
| G1 | 5.21 | 0.98 | 2317.65 | 2456.02 | 1854 | 0.69 | 68478 | 3.22 | 0.94 | 63.25 | 76.34 | 37 | 0.89 | 90 | 3.98 | 0.97 | 152.31 | 153.90 | 106 | 0.85 | 636 |
| G2 | 4.77 | 0.97 | 1833.53 | 1927.04 | 1490 | 0.65 | 60524 | 2.81 | 0.90 | 72.75 | 73.57 | 30 | 0.83 | 84 | 3.40 | 0.93 | 113.07 | 120.45 | 73 | 0.79 | 332 |
| P1 | 5.19 | 0.98 | 2448.08 | 2555.45 | 1864 | 0.69 | 52399 | 3.07 | 0.94 | 48.50 | 47.32 | 31 | 0.89 | 84 | 3.87 | 0.96 | 146.65 | 154.64 | 91 | 0.86 | 338 |
| P2 | 5.07 | 0.98 | 1465.24 | 1590.43 | 1233 | 0.71 | 27475 | 2.63 | 0.91 | 29.25 | 33.49 | 18 | 0.91 | 38 | 3.52 | 0.95 | 81.27 | 81.93 | 54 | 0.88 | 166 |
| P3 | 5.06 | 0.98 | 2220.37 | 2322.60 | 1830 | 0.67 | 77010 | 2.94 | 0.91 | 90.50 | 74.23 | 38 | 0.81 | 144 | 3.80 | 0.95 | 131.12 | 138.37 | 103 | 0.82 | 478 |
| P4 | 5.05 | 0.98 | 1745.00 | 1868.08 | 1491 | 0.69 | 53546 | 3.05 | 0.93 | 52.13 | 57.36 | 33 | 0.87 | 83 | 3.67 | 0.95 | 120.00 | 128.56 | 85 | 0.83 | 351 |
| P5 | 4.83 | 0.97 | 1612.51 | 1721.61 | 1326 | 0.67 | 44641 | 2.85 | 0.91 | 44.14 | 56.85 | 27 | 0.86 | 62 | 3.43 | 0.93 | 84.00 | 99.48 | 63 | 0.83 | 224 |
| P6 | 4.82 | 0.97 | 1442.70 | 1559.58 | 1197 | 0.68 | 34257 | 2.79 | 0.91 | 49.25 | 64.72 | 23 | 0.89 | 46 | 3.51 | 0.95 | 89.83 | 101.32 | 56 | 0.87 | 157 |
| R1 | 5.27 | 0.98 | 2230.36 | 2298.98 | 1830 | 0.70 | 81329 | 3.02 | 0.94 | 56.20 | 58.65 | 29 | 0.90 | 71 | 3.97 | 0.97 | 113.11 | 123.67 | 87 | 0.89 | 382 |
| R2 | 5.04 | 0.98 | 1855.07 | 1941.99 | 1515 | 0.69 | 40556 | 3.04 | 0.94 | 47.00 | 47.55 | 26 | 0.93 | 50 | 3.83 | 0.97 | 134.58 | 129.05 | 85 | 0.86 | 420 |
| R3 | 4.92 | 0.97 | 2223.48 | 2331.37 | 1686 | 0.66 | 50608 | 2.94 | 0.93 | 46.00 | 60.78 | 26 | 0.90 | 53 | 4.02 | 0.97 | 145.15 | 131.95 | 102 | 0.87 | 592 |
| R4 | 4.95 | 0.98 | 1833.78 | 1956.36 | 1456 | 0.68 | 40530 | 2.71 | 0.91 | 29.00 | 34.86 | 20 | 0.90 | 46 | 3.83 | 0.97 | 104.07 | 105.13 | 79 | 0.88 | 383 |
| R5 | 5.13 | 0.98 | 2466.18 | 2539.69 | 1821 | 0.68 | 43591 | 2.88 | 0.93 | 31.50 | 35.10 | 24 | 0.91 | 68 | 3.92 | 0.97 | 152.65 | 158.55 | 97 | 0.86 | 402 |
| S1 | 5.09 | 0.98 | 1869.19 | 1952.62 | 1482 | 0.70 | 48880 | 2.97 | 0.92 | 44.13 | 52.89 | 31 | 0.87 | 90 | 3.62 | 0.95 | 87.25 | 94.35 | 70 | 0.85 | 320 |
| S2 | 5.09 | 0.98 | 2104.83 | 2136.06 | 1682 | 0.69 | 55472 | 3.41 | 0.95 | 89.25 | 125.18 | 42 | 0.91 | 79 | 3.70 | 0.95 | 112.00 | 110.72 | 81 | 0.84 | 371 |
| S3 | 5.05 | 0.98 | 2043.24 | 2106.85 | 1654 | 0.68 | 64437 | 3.09 | 0.93 | 42.27 | 49.85 | 34 | 0.88 | 103 | 3.64 | 0.95 | 121.06 | 126.27 | 88 | 0.81 | 458 |
| S4 | 4.99 | 0.98 | 1209.08 | 1284.12 | 1051 | 0.72 | 26399 | 2.91 | 0.92 | 49.67 | 63.25 | 27 | 0.88 | 60 | 3.72 | 0.96 | 96.00 | 91.85 | 69 | 0.88 | 247 |
| S5 | 5.13 | 0.98 | 1773.05 | 1933.83 | 1425 | 0.71 | 26175 | 2.32 | 0.89 | 14.00 | 15.94 | 12 | 0.93 | 25 | 3.87 | 0.97 | 94.89 | 106.07 | 72 | 0.90 | 213 |
| T1 | 5.10 | 0.98 | 1413.37 | 1533.16 | 1056 | 0.73 | 14601 | 2.57 | 0.89 | 43.00 | 68.60 | 17 | 0.91 | 27 | 3.62 | 0.95 | 111.00 | 110.18 | 60 | 0.88 | 150 |
| T2 | 5.12 | 0.98 | 1712.30 | 1741.44 | 1291 | 0.71 | 24146 | 2.66 | 0.91 | 31.75 | 35.80 | 18 | 0.92 | 34 | 3.70 | 0.96 | 92.24 | 101.02 | 70 | 0.87 | 253 |
| T3 | 5.06 | 0.98 | 1228.29 | 1315.87 | 953 | 0.74 | 11588 | 2.48 | 0.90 | 36.50 | 33.81 | 14 | 0.94 | 22 | 3.68 | 0.96 | 101.06 | 127.46 | 64 | 0.88 | 156 |
| T4 | 5.05 | 0.98 | 1097.89 | 1157.77 | 865 | 0.75 | 10211 | 2.86 | 0.94 | 58.00 | 90.00 | 18 | 0.99 | 20 | 3.38 | 0.95 | 59.55 | 59.36 | 44 | 0.89 | 119 |
| U1 | 5.27 | 0.98 | 1663.96 | 1760.08 | 1269 | 0.74 | 20958 | 2.71 | 0.92 | 35.50 | 40.82 | 19 | 0.92 | 35 | 3.99 | 0.97 | 156.91 | 131.44 | 86 | 0.90 | 278 |
| U2 | 5.20 | 0.98 | 1778.73 | 1875.10 | 1404 | 0.72 | 27152 | 2.89 | 0.92 | 42.50 | 56.83 | 25 | 0.90 | 53 | 3.78 | 0.96 | 123.06 | 128.26 | 86 | 0.85 | 365 |
| U3 | 5.18 | 0.98 | 1684.14 | 1810.26 | 1253 | 0.73 | 18628 | 2.93 | 0.94 | 48.25 | 47.92 | 22 | 0.95 | 35 | 3.86 | 0.97 | 97.89 | 106.64 | 75 | 0.89 | 248 |
| U4 | 5.00 | 0.98 | 1459.63 | 1511.33 | 1042 | 0.72 | 15113 | 2.40 | 0.90 | 16.20 | 19.64 | 12 | 0.97 | 18 | 3.70 | 0.96 | 109.15 | 113.01 | 66 | 0.88 | 200 |
| U5 | 5.26 | 0.98 | 1401.36 | 1493.90 | 1178 | 0.74 | 20178 | 2.53 | 0.88 | 36.50 | 80.31 | 17 | 0.89 | 27 | 3.62 | 0.96 | 105.00 | 144.95 | 60 | 0.88 | 144 |
